# A comparative study of deep eutectic solvents based on fatty acids and the effect of water on their intermolecular interactions

**DOI:** 10.1038/s41598-023-50766-1

**Published:** 2024-01-19

**Authors:** Samaneh Barani Pour, Jaber Jahanbin Sardroodi, Alireza Rastkar Ebrahimzadeh, Gholamreza Pazuki, Vahideh Hadigheh Rezvan

**Affiliations:** 1 Molecular Science and Engineering Research Group (MSERG), Molecular Simulation Lab, Azarbaijan Shahid Madani University, Tabriz, Iran; 2 Molecular Science and Engineering Research Group (MSERG), Department of Chemistry, Molecular Simulation Lab, Azarbaijan Shahid Madani University, Tabriz, Iran; 3 Molecular Science and Engineering Research Group (MSERG, Department of Physics, Molecular Simulation Lab, Azarbaijan Shahid Madani University, Tabriz, Iran; 4 Department of Chemical Engineering, Amirkabir University of Technology, Tehran, Iran; 5 Department of Chemistry, Ardabil Branch, Islamic Azad University, Ardabil, Iran

**Keywords:** Chemistry, Engineering, Mathematics and computing

## Abstract

In this work, intermolecular interactions among the species of fatty acids-based DESs with different hydrogen bond acceptors (HBA) in the adjacent water have been investigated using molecular dynamics (MD) simulation. The results of this work provide deep insights into understanding the water stability of the DESs based on thymol and the eutectic mixtures of choline chloride and fatty acids at a temperature of 353.15 K and atmospheric pressure. Stability, hydrogen bond occupancy analysis, and the distribution of the HBA and HBD around each other were attributed to the alkyl chain length of FAs and the type of HBA. Assessed structural properties include the combined distribution functions (CDFs), the radial distribution functions (RDFs), the angular distribution functions (ADFs), and the Hydrogen bonding network between species and Spatial distribution functions (SDF). The reported results show the remarkable role of the strength of the hydrogen bond between THY molecules and fatty acids on the stability of DES in water. The transport properties of molecules in water–eutectic mixtures were analyzed by using the mean square displacement (MSD) of the centers of mass of the species, self-diffusion coefficients, vector reorientation dynamics (VRD) of bonds and the velocity autocorrelation function (VACF) for the center of the mass of species.

## Introduction

The interface between water and liquids is one of the most important factors in the separation process. The structural and dynamical information of molecules at the water/liquid interfaces has drawn much interest as a heterogeneous field for various chemical reactions. The characterization of the water/DESs interface can be useful because of the important role of DESs in dissolving material compounds and separation processes. In various industrial settings, volatile organic compounds (VOCs) have important uses as solvents. The organic solvents are reported to cause various respiratory diseases and cancer^1^. Therefore, ionic liquids (ILs) are suggested as alternative solvents to ensure human health^2^. There have been reports on ionic liquids such as the negligible volatility and non-flammability have been suggested as “green” solutions^2^. Compared to ionic liquids, DESs have received attention because of cheaper to make, much less toxic, and biodegradable^3^. Eutectic mixtures are the ‘green’ solvents that have a melting point much lower than that of either of their components^4^. The deep eutectic solvent was first introduced by Abbot et al. (2003) for the binary mixture of urea and choline chloride^5^.

DES-based on Hydrophilic compounds have received attention in various applications the desulfurization of fuel oil and natural gas and synthesis of nanomaterials, and electrochemistry^6^. Furthermore, the potential of these solvents in natural gas sweetening for CO_2_ capture is impressive^7^. However, be recognized that in the case of mixtures containing benzene and thiophene, monoethanolamine-based deep Eutectic solvents as the extraction solvents do play a significant role in the separation of species^8^.

In an aqueous environment, hydrophilic DESs often easily dissolved in water because of their hydrogen-bonding ability^9^.The stability of the hydrophobic deep eutectic solvents (HDESs) in water has widened their application field in the aquatic environment. Eutectic mixtures based on the decanoic acid and quaternary ammonium salt were introduced as HDESs for the first time^10^. The long chain length of the two HBA and HBD components led to the lower solubility of these systems in water (The solubility of the decanoic acid in water is approximately 0.15 mg g^−1^ at 20 °C.20 A)^10^. It has been previously reported that DESs based on straight-chain monobasic acids and choline chloride is beneficial for the extraction of the sulfur compounds from model oil^11^. Recently, DESs consisted of longer-chain fatty acids, and choline chloride was introduced^12^. The hydrophobic properties of the deep eutectic solvents are dependent on two components hydrogen bond donor (HBD) and hydrogen bond acceptor (HBA) can be easily tailored. The stability of the choline chloride -fatty acids acid mixture is negligible in aqueous solutions. Therefore, replacing terpenes with ammonium salts in the structure of DES can increase the hydrophobic properties of the eutectic solvent and expand the application of solvents in the extraction process. Hydrophobic eutectic solvents based on terpenes and monocarboxylic acids are relatively insoluble in aqueous solutions. The researchers found that the Hydrophobic DESs based on DL-menthol with fatty acids can be applied for the extraction of neonicotinoids from diluted aqueous solutions^13^.

In this work, the stability of the eutectic mixtures based on fatty acids and choline chloride in the aqueous environment was investigated. Due to the wide application of these solvents in the industry, theoretical and simulation techniques have been used to shed light on the behavior of DES in contact with water. The molecular dynamics (MD) simulation is a suitable technique for understanding these interfacial phenomena^6^. The molecular-level understanding DESs in the adjacent water are necessary for further investigations of the stability of the eutectic mixtures. In addition, the performance of DES can be optimized in the separation process by taking into account the water/DES interface.

Short range van der Waals and electrostatic interactions, and hydrogen bonding were used to the describing intermolecular interaction in the pure DESs and solutions of DES and water. The present study involves the stability of DESs based on fatty acids in water at a temperature of 353 K and 1 atm pressure by using MD simulation. The ‘relative stability factor’ and the structural properties were evaluated to get valuable insights into the stability of DESs in water. The analysis shows fewer non-bonding interactions between adjacent water molecules and thymol-based DESs. According to the relative stability factor, the approximate order of stability of DES in adjacent water is as follows:

Choline chloride: Acetic acid (1:1) < Choline chloride: Butyric acid (1:1) < Choline chloride: Caprylic acid (1:1) < Choline chloride: Decanoic acid (1:1) < Choline chloride: Myristic acid (1:1) < Thymol: Acetic acid (1:1) < Thymol: Butyric acid < Myristic acid (1:1) < Thymol: Decanoic acid (1:1) < Thymol: Myristic acid (1:1).

## Methodolog

### COSMO-RS analysis

**Figure 1:**
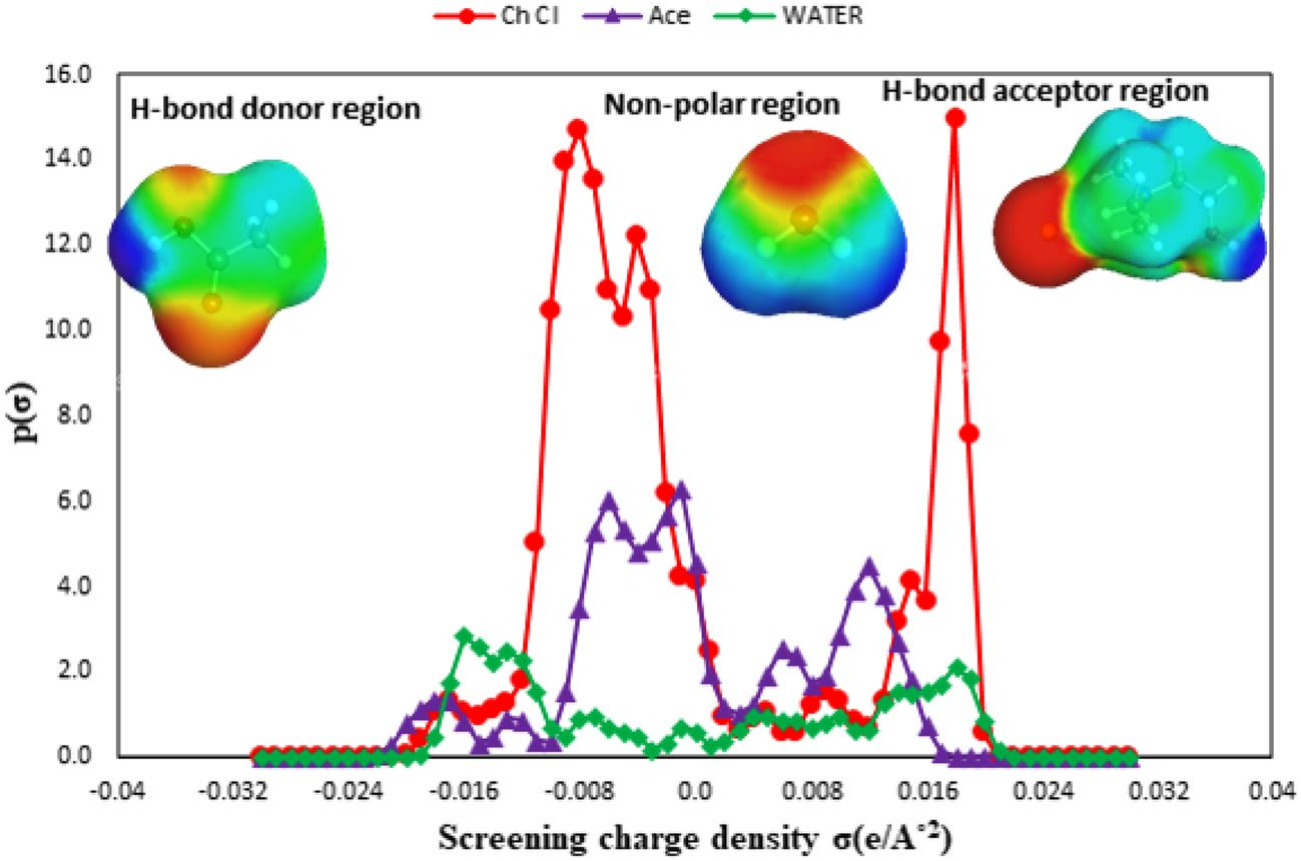
Sigma—profiles of Choline chloride (red), Acetic acid (purple), and water (green) and their sigma-surfaces representation.

Conductor-like Screening Model for Real Solvents (COSMO-RS) parameters are thermodynamic models based on quantum chemistry and statistical thermodynamics^13^. To reflect the strength of the interactions between HBA (choline chloride or thymol) and HBD (Fatty acids) in the eutectic solvents, sigma-surface, and sigma-profile were predicted using COSMO-RS. In order to, the individual molecules of the eutectic solvents are optimized using the Density Functional Theory (DFT) BP level and triple-zeta valence potential (TZVP) basis set^14^. Before analysis of the molecular interactions between HBA and HBD, the polarity properties of species of the eutectic mixtures are predicted using COSMO-RS. Three divisions related to σ-profile are as follows: (1). Hydrogen bond donor region (HBD) is at $-\infty <\sigma < -0.0082 [e{A}^{-2}]$, (2). Non-polar region exist at $-0.0082<\sigma < + 0.0082 [e{A}^{-2}]$, (3). Hydrogen bond acceptor region (HBA) is at $\sigma < -0.0082 [e{A}^{-2}]$^15^. In Figs. 1 and S14 positive values of the sigma-profile plot between 0.0082 and 0.032 e/Å^2^ correspond to the presence of hydroxyl groups and Cl^−^ anion. On the other, the HBD region has negative values of sigma-profile that are between − 0.032 and − 0.0082 e/Å^2^ and show hydrogen atoms in molecules. Also, the strong peak of non-polar regions (− 0.0082 < σ < 0.0082 e/Å^2^) is characteristic of the aromatic ring and alkyl chain (the neutrally charged zone). Sigma surfaces of the species of eutectic solvents are shown in the upper part of Figs. 1 and S14. In the sigma surface, red regions correspond to the negatively charged atoms (O atom), green is carbon atoms that have neutral charge density, and blue zones to the positively charged atoms (H atom).

### MD Simulation

Molecular dynamics simulations were carried out for studying the stability of the DESs-based on fatty acids in water using the NAMD_2.13 package using a 12 Å cut-off distance for Van der Waals interactions (*Lennard–Jones*, LJ). The binary mixtures of HBD and HBA were randomly prepared using the PACKMOL package^16^. Force field parameters (force constants for bonds and angles and non-bonded parameters) and the partial atomic charges for the fatty acids were not available, so charge parameters were obtained at the MP2/6-31G* level by fitting the RESP (restricted electrostatic potential). More details about simulation parameterization were reported in our previous work^17^. The TIP3P model was used for water simulation. The particle mesh Ewald method was used to calculate the Electrostatic interactions and the cutoff distance for the van der Waals terms was set to be 12 Å^18^. Periodic boundary condition (PBC) was applied during the simulation and the time step of 2 fs was considered^19^. Initially, all the systems were minimized to remove bad atomic contacts. In the next step, the binary mixtures were heated to 353 K temperature. Finally, equilibration runs were performed for 30,000,000 steps in NPT ensembles i.e., with constant temperature, pressure, and the number of particles at 353 K. Langevin algorithm was applied for keeping the temperature and the pressure periodic boundary constant (P = 1 atm and T = 353 K). The last 20 ns of the simulation were analyzed with Visual Molecular Dynamics (VMD). The equations of motion were solved using Verlet algorithm and a time step of 1 fs^20^.

**Figure 2:**
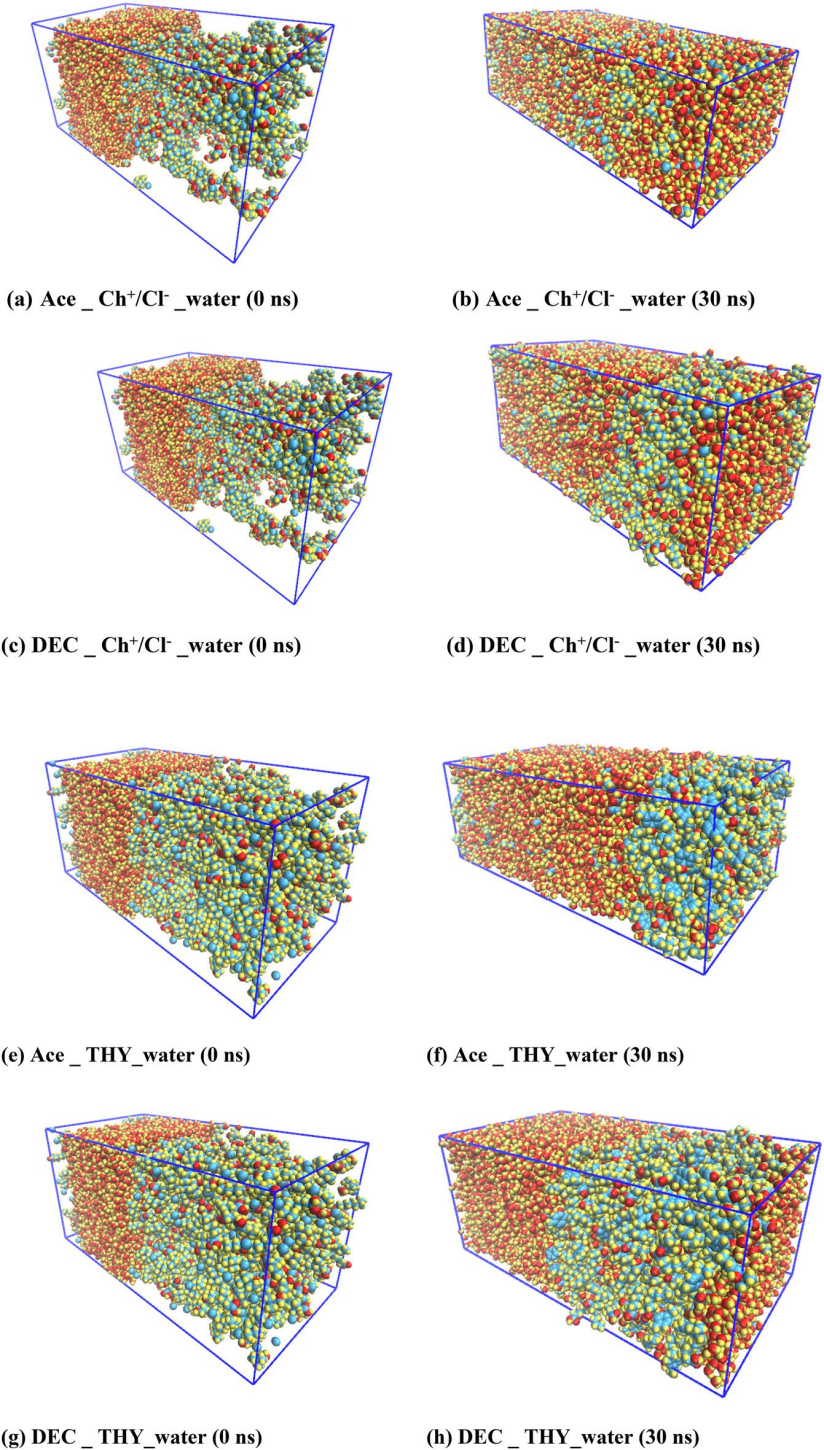
Distribution snapshots of the DES—water systems in equilibrium at the last 20 ns of MD simulation for (**a**, **d**) the binary mixtures of [Ch^+^][Cl^−^] and FAs, (**e**, **h**) the binary mixtures of THY and FAs.

The structural and dynamic properties of the binary mixtures in equilibrium were investigated. In addition, the water molecules were packed in another cubic box. The two cubic boxes merged together to get a rectangular box with z dimension (We will refer to this as the binary systems in the adjacent water). Similar to eutectic solvent at the Pure State, its dynamic and structural properties were analyzed at 353 K. The binary systems contain the same amount of water and the binary systems with the constant molar ratio of THY: FAs and [Ch^+^][Cl^−^]: FAs = 1:1. Figure 2 shows the system configuration in the first simulation and after systems have reached equilibrium. All the structures were analyzed at 353 K. The structural properties of the binary systems were investigated using hydrogen bonds, spatial distribution functions (SDF) and combined distribution functions of the angular distribution functions (ADFs) and the radial distribution functions (RDF). Finally, to analyze the stability of fatty acids-based DESs the relative stability factor was introduced in the aquatic environment. The binary mixtures were solvated in a water box (total number of water molecules: 750,000). Names, terpenes, and fatty acids understudy and abbreviations for the binary mixtures are listed in Table 1.

**Table 1:** Names of the simulated eutectic solvents and their different combinations (HBD and HBA) with abbreviations in the binary mixtures.

Name	HBD	HBA	Molar ratio	n HBA : n HBD
CAC	Acetic acid (Ace)	Choline Chloride (Ch^+^ Cl^−^)	1:1	500:500
CBU	Butyric acid (BUA)	Choline Chloride (Ch^+^ Cl^−^)	1:1	500:500
CDA	Decanoic acid (DEC)	Choline Chloride (Ch^+^ Cl^−^)	1:1	500:500
CMA	Myristic acid (MRA)	Choline Chloride (Ch^+^ Cl^−^)	1:1	500:500
TAC	Acetic acid (Ace)	Thymol (THY)	1:1	500:500
TBU	Butyric acid (BUA)	Thymol (THY)	1:1	500:500
TDA	Decanoic acid (DEC)	Thymol (THY)	1:1	500:500
TMA	Myristic acid (MRA)	Thymol (THY)	1:1	500:500

## Results and discussion

### Structure analysis

Microscopic structural analysis of pure deep eutectic solvents and aqueous DES solutions helps to understand the unique properties of these DESs. The hydrogen bond network between the hydrogen-bond donor (HBD), and hydrogen-bond acceptor (HBA) perturbed in the adjacent water, results in altering the physical properties of the importance^21^. Fig. S1 shows labels for the atoms in the different acids as the hydrogen bond donors.

### Radial distribution functions

**Figure 3:**
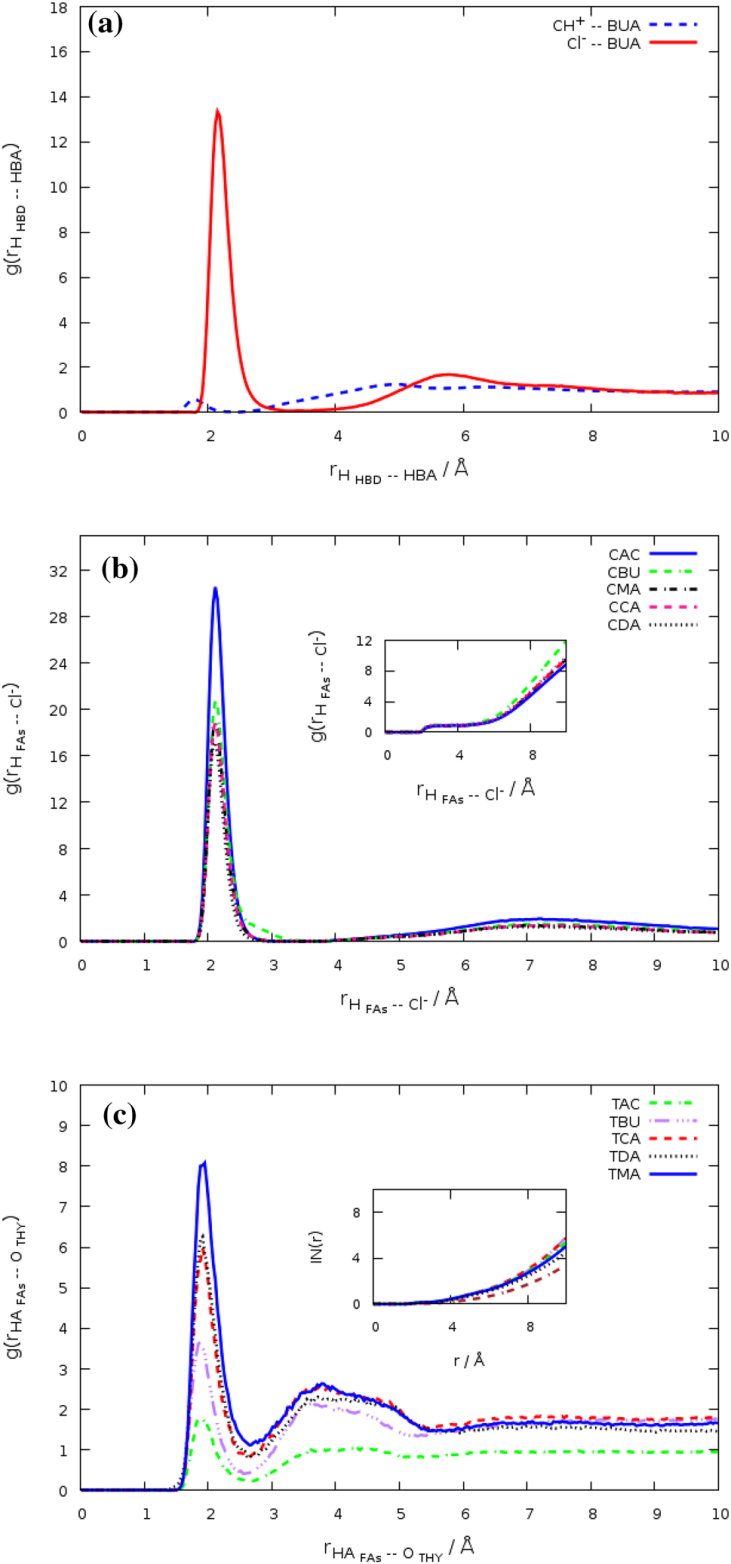
(**a**) RDFs between DEC molecules and choline chloride salt, ${({\text{gr}})}_{{Ch}^{+}-FAs}$, ${{\text{g}}({\text{r}})}_{{Cl}^{-} -\mathrm{ FAs}}$, for the binary mixture at 353 K. (**b**) RDF between the HA atom of FAs and Cl^−^ anion for the binary mixtures at 353 K. (**c**) RDFs between HBD and HBA,${{\text{g}}({\text{r}})}_{\mathrm{HBD }-\mathrm{ HBA}}$, for the binary mixture at the adjacent water.

The radial distribution function (RDF), also known as $g(r)$, provides insight into the structure of the binary mixture. This function and coordination numbers can be used to check the distribution of species around each other. The coordination numbers show the number of molecules within the first solvation shell of RDFs^21^. RDFs are computed by the following equation:

1 $$ g\left( r \right) = \frac{1}{\rho N}\left\langle {\mathop \sum \limits_{ij} \delta \left( {r - r_{ij} } \right)} \right\rangle $$

where N depicts the number of particles in the system, $\rho$ is number density. We characterized the closest atoms of different molecules by computing the atom–atom radial distribution functions (RDFs). These atomic sites are the HA and O atoms of FAs and the O and HA atoms of THY molecules. RDF between the different atoms FAs and HBA for the fatty acids based deep eutectic solvents is shown in Fig. S2. RDFs between HBA and HBD were analyzed to characterize the structural correlation in aqueous environments. In each of the systems, the H atom of the carboxylic acid of FAs molecules is surrounded by the chloride anion. The Cl^−^–FAs RDFs and RDFs between choline cations and FAs results suggest that H-bonds interaction between the cation and FAs is very weak than FAs–Cl^−^ interaction (see Fig. 3a). However, the hydroxyl group and N_CHL_ of the choline seem to be involved in the coordination of the benzene molecule in the DESs based on choline chloride and monoethanolamine^8^. Furthermore, the distribution of anions around the HA atom of Ace molecules was much more impressive than the very long-chain fatty acids (see Fig. 3b). The first peak in the radial distribution function, $g({r}_{O2-HA})$, shows the formation of hydrogen bonds between the highest electronegative atoms (O) of THY and the H atom bonded to FAs molecules. Electrostatic interactions are the effective factor in the hydrogen bond formation between the HA atom of FAs and anions (see Fig. S3). The $g\left( r \right)_{FAs - THY}$ have a main peak and a small plateau in the vicinity of the first RDF maximum, while RDFs of THY molecules around each other have a shoulder at 2.5 Å. We focused on the effect of water on the interaction of two species in a binary mixture. RDFs of HBD around HBA were analyzed in the pure state and adjacent water at 353 K. The first maximum of the RDFs between choline chloride salt and FAs shows the probability of finding Cl^−^ anion within a certain radius around the COOH group of FAs. It should be noted that the sharp peak of the RDF has been significantly reduced in the adjacent water. Most likely, reducing the height of the peak can be related to the interaction between salt and water in the ternary mixtures. The HBA–FAs RDF for the binary mixtures of [Ch^+^][Cl^−^] and fatty acids, as shown by ${g\left(r\right) }_{{Cl}^{-}-FAs}$, the first solvation shell of ${g\left(r\right) }_{{Cl}^{-}-FAs}$ are clearly observed at 2 $A^\circ$. The sharp peak of ${g\left(r\right) }_{{Cl}^{-}-FAs}$ indicates the formation of the H-bond between the two species in the binary mixtures. The species distribution around each other was analyzed in detail by coordination number (CN). The coordination number can be found through the numerical integration of the function g(r) up to its first minimum^22^. The coordination number were calculated from RDFs on the basis of the distance r from the center-of-mass (COM) the reference molecule. Integration of the RDF of Cl^−^ anion around FAs molecules has the number of anions of 0.1485 in the binary mixture of MRA and [Ch^+^][Cl^−^] salt. The Ace–Cl^−^ RDF shows slightly higher coordination with the 0.495 values at 353 K. It should be noted that the number of cations under the first peak of the RDF between FAs and choline cations is very little. Coordination numbers are obtained near 0.1656 for the BUA … TIP3 pair and 1.2679 for the Cl^−^ … TIP3 pair in the binary mixtures. RDFs of the water molecules around species of the Ch^+^/ Cl^−^ salt and FAs mixtures have two peaks at 2 Å and 3 Å. However, the second peak of ${g(r)}_{TIP3-Salt}$ be clearly seen, whereas the ${g(r)}_{TIP3-FAs}$ show the opposite behavior. The second peak of RDF between choline chloride and water molecules confirms the long-range electrostatic interactions in the binary systems^23^. The first and second solvation shells of RDF between THY molecules are clearly observed at 2 and 3 Å that the RDF height decreased in the presence of FAs with the shorter chain length acids. The intensities of RDF peaks between species of the eutectic solvents were investigated in states pure and in the adjacent water. The comparative analysis of the RDFs between species of DESs and water molecules is shown in Figs. S4 and S5. We calculated the RDF between the H atoms of water molecules around the hydroxyl group of FAs molecules. The wide peak of ${g(r)}_{ TIP3 - {O}_{FAs}}$ indicated the weak interaction between FAs molecules and water molecules in the binary mixtures. The overall structural properties of [Ch^+^/Cl^−^] [FAs] and [THY][FAs] mixtures were compared at 353 K. The comparative analysis of the RDFs between HBD and HBA of the binary mixtures in the adjacent water is shown in Fig. 3c). Similarly, with the binary mixture of [Ch^+^/Cl^−^ salt and FAs, the sharp peak of RDF between the HA atom of FAs and the O atom of THY molecule is located at 2 Å for the binary mixtures of THY and FAs. In fact, the distribution of the HA atom of FAs molecules around the electronegative atoms (O) of THY molecules separates the first solvation shell in the binary mixtures of THY and FAs. In addition, the sharp peak of ${g(r)}_{TIP3-THY}$ can be seen at 2 Å, similar to the first solvation shell of RDF between chloride anion and water molecules in the binary mixtures. According to Fig. 2, the tendency of species to the water-rich phase is impressive in the binary mixtures with the shorter chain length acids. The peak height of RDFs between the different species is indistinguishable so the coordination number was calculated in different systems. The number of water molecules under the first peak of ${{g(r)}_{TIP3-[{Ch}^{+}] [{Cl}^{-}]}}$ and ${g(r)}_{TIP3 - FAs}$ are 1.2328 and 0.2184 for [Ch^+^][Cl^−^] and Ace. The variation of coordination number with respect to Fatty acids in their pure state and CN of chloride anions around the atoms of species are listed in Table. S1. The RDF between the mass centers of HBA and HBD has a coordination number of 0.0095 in the binary mixtures of Ace and Choline chloride in the adjacent water at 353 K. The water molecules lead to much lower coordination numbers between HBA and HBD. The coordination number of HBA–HBD RDF of binary mixtures containing water and DES is less than the CN of RDF between [Ch^+^][Cl^−^] and Ace in the binary mixtures at a pure state.

The strong interaction between the choline chloride salt and Acetic acid can be clearly at 353 K (see Table. S1). This interaction is most likely related to the aggregation of anions around the HA atom of acid molecules. The distribution of anions around the HA atom compared to other acid atoms has been discussed earlier in the above. The aggregation degree of HBD around HBA was reduced for the very long-chain fatty acids, due to the weakening of their interactions. Intermolecular hydrogen bonds are involved in the formation of eutectic solvents so the combined radial/angular distribution functions were computed in this work. The aim of this analysis is the further explore the distances and angles to determine hydrogen bonding.

**Figure 4:**
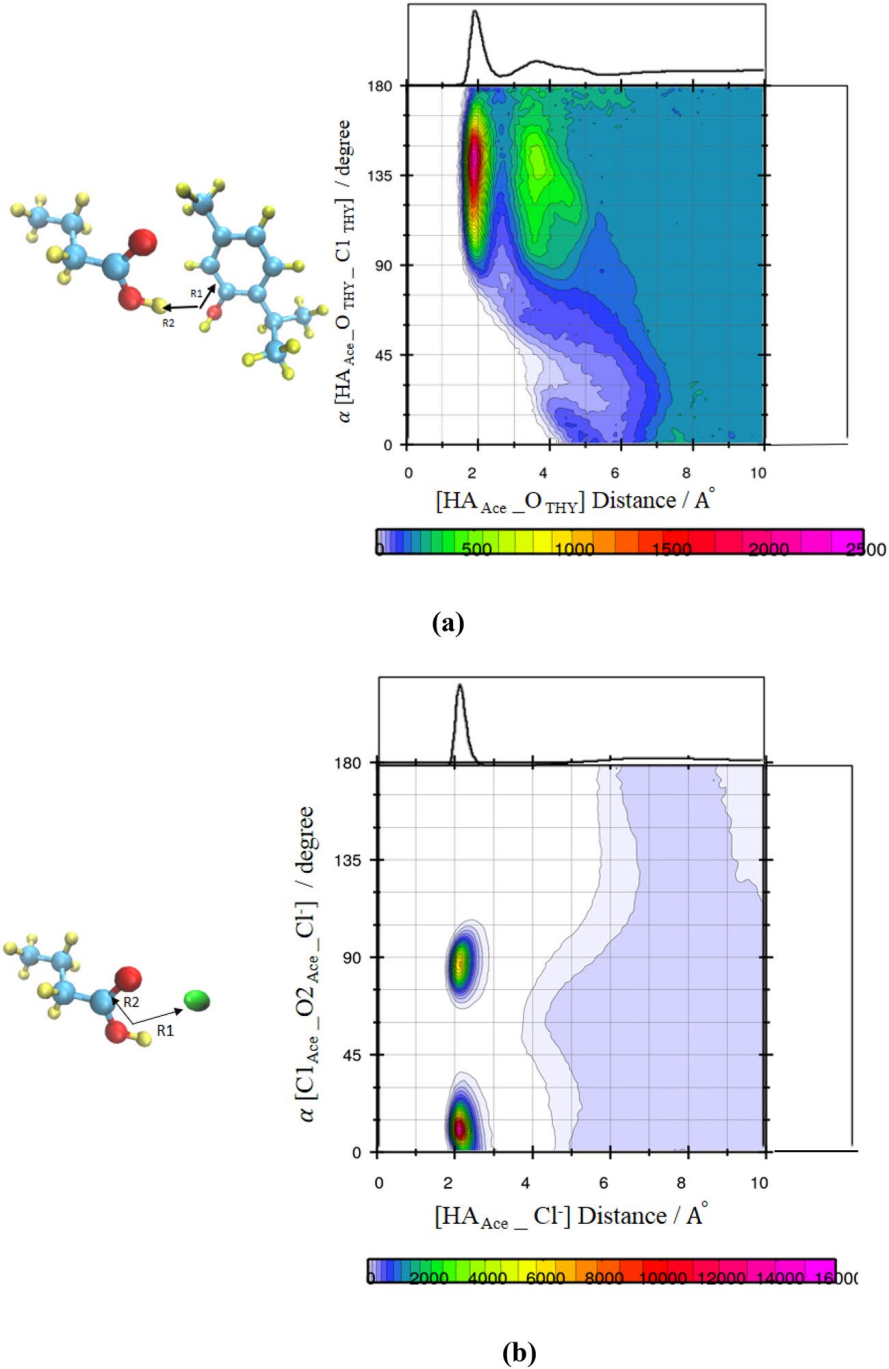
Combined radial/angular distribution functions for (**a**) the HA Ace_O_THY_ distance and HAAce_OTHY_C1 _THY_ angle, (**b**) the HA _Ace__Cl^−^ distance and C1_Ace_–O2_Ace_–Cl^−^ angle.

**Figure 5:**
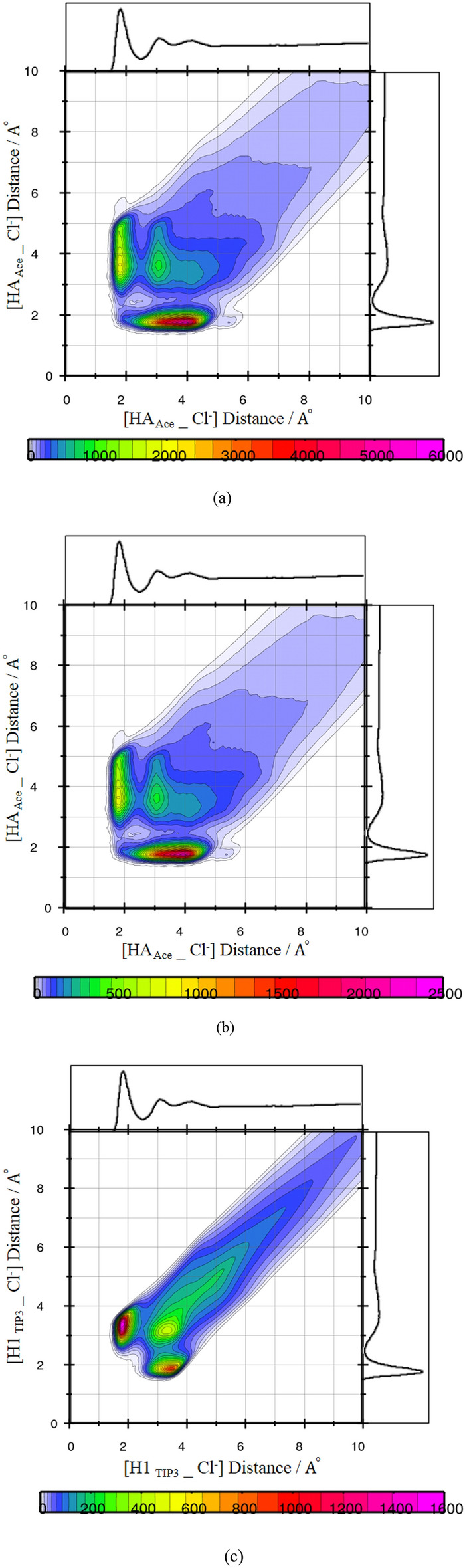
Combined radial/radial distribution function between Cl^−^ anion and Ace molecules in the binary mixtures at pure state (**a**) Combined radial/radial distribution function between Cl^−^ anion and Ace molecules in the binary mixtures at the adjacent water, (**b**) Combined radial/radial distribution function between Cl^−^ anion and water molecules in the binary mixtures (**c**).

The respective CDF includes the two axes that X-axis illustrates the distance between the H atoms of the FAs, HA, and Hydroxyl oxygen of the neighbor THY, O, and the angle between two vectors (R1 and R2) of the fatty acid-thymol neighbors as the Y-axis. According to the curve, the geometric conditions of H-bond formation for THY–FAs structural correlation was estimated at the region around 135°–150°/2 Å (see Fig. 4a). The H-bond distance criteria were determined using the first minimum of the corresponding RDFs and the angular criteria were obtained from the probability region in the angular/distance CDFs. The angles/distance range at 130 and180°/2–3 Å from CDFs was considered for H—bond analysis in the binary mixtures of FAs and [Ch^+^][Cl^−^] (see Fig. 4b). The possibility of the interaction of two species HBA and HBD were examined by using the CDFs of the combined RDF between the HA atom of HBD and the electronegative atoms of HBA $, g({r}_{{HA}_{FAs} - {Cl}^{-}} )$. The composed CDFs of the RDF between the HA atom of the carboxyl group of FAs and Cl^−^ anion ADF between the R1 and R2 vectors (R1 and R2 shown in Fig. 4a) were plotted in the adjacent water and the pure state (see Figs. S6 and S7). The preferred water-anion (H_TIP3_-Salt) H-bond rather than the HBD-HBA (H_FAs_–Cl^−^) H-bond is confirmed by the distance/distance CDFs. The water molecules as one bridge can accept H-bonds using the O atom and donate O–H to chloride anion and Ch^+^ cation. Compared to choline chloride salt, if there is a possibility of interaction between water molecules and thymol molecules, it is very insignificant (see Fig. 5a–c).

### Hydrogen-bond analysis

**Figure 6:**
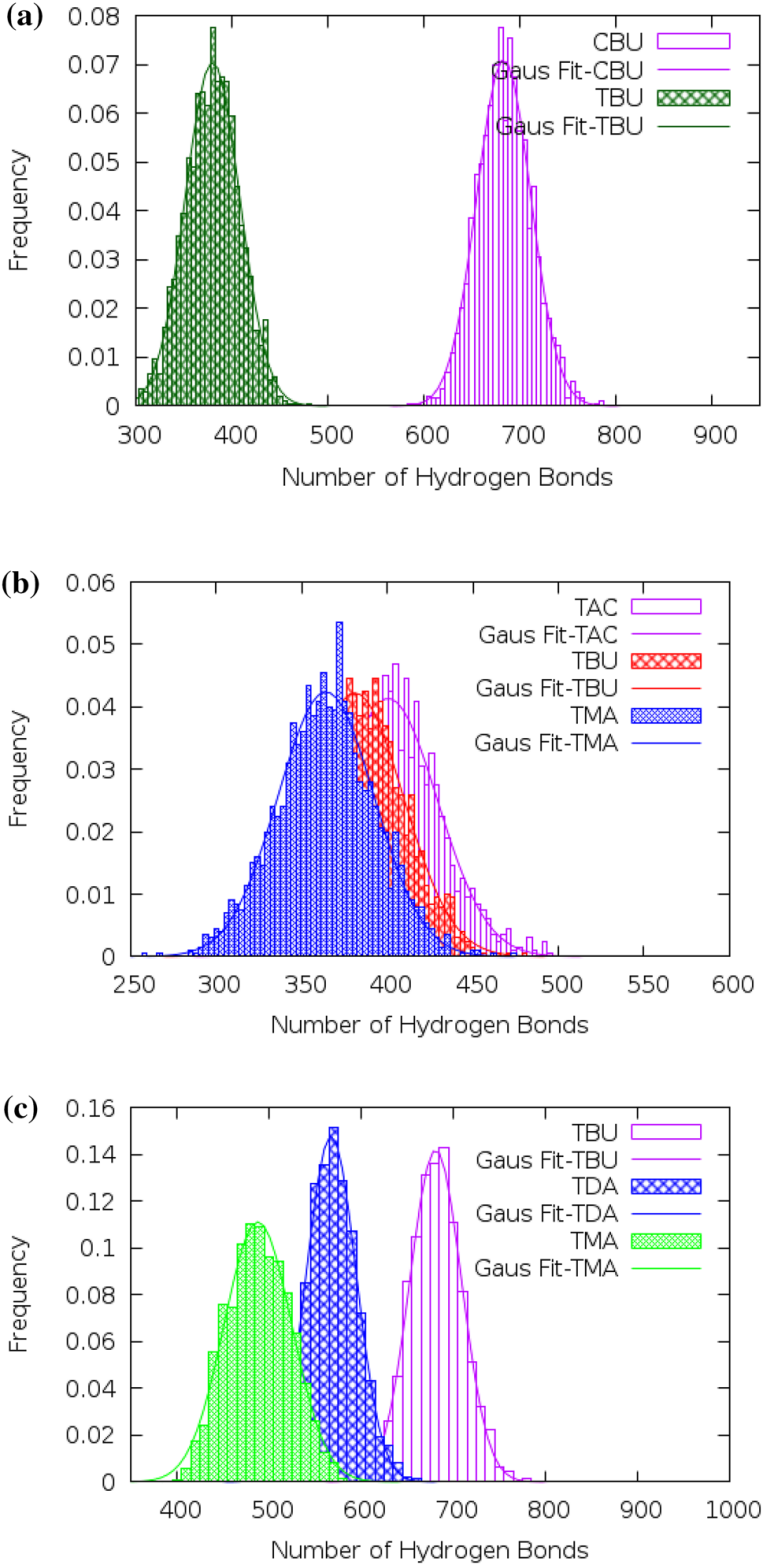
(**a**) The distribution of the hydrogen bond between the HA atom of BUA and HBA at 353 K. (**b**) The distribution of the hydrogen bond between the HA atom of FAs and THY molecules at 353 K. (**c**) The distribution of the hydrogen bond between the HA atom of FAs and Cl^−^ anion at 353 K.

Intermolecular hydrogen bonds (HB) play an important role in the stability of eutectic solvents in an aqueous solution. It should be noted that the weaker binding ability of HBD to HBA reduced the hydrogen bond network in DES which plays an important role in the extraction processes^24^. Criteria for the identification of hydrogen bonds were determined by the Combined RDF and ADF Analysis. According to the CDF analysis, hydrogen bonds should be defined using the angles cutoff range 135°–150° and the $D.H... A$ distance less than 3.5 Å [hydrogen donor atoms (D) and the hydrogen acceptor atom (A)] in the binary mixtures of FAs and Thymol^25^. The fraction of H-bonds interactions between the different atoms of HBA ([Ch^+^][Cl^−^] and THY molecules) and the Hydroxyl group of HBD was investigated in the binary mixtures at the pure state. There is a relative contribution of hydrogen bonds between the HA atom of HBD molecules and anion of [Ch^+^][Cl^−^] (the O atom of THY) which is more than other atoms of HBA species. The number of hydrogen bonds plotted over time in the binary mixtures for the last 20 ns of MD simulation. The average number of H-bonds were determined by fitting the distribution histograms with the Gaussian function (Eq. 2).

2 $$F\left(X\right)={\frac{a}{\sigma \sqrt{2\pi }}exp}^{\frac{{-(X-\overline{X })}^{2}}{2{\sigma }^{2}}}$$

where $\overline{X }$ is the average number of H-bonds that are discussed follow. The $a$ and $\sigma$ are the adjustable parameter, and standard deviations, respectively^26^. Figure 6a shows the distribution of the number of H-bond between HBA and HBD in the binary mixtures at 353 K. The average number of H-bond between anion of [Ch^+^][Cl^−^] and the Butyric acid molecule is 684.438 ± 0.5573 in the binary mixture. As shown in Table. S2, the maximum number of H-bond obtained between HBA and HBD in the binary mixture of BUA molecules and choline chloride is significant compared to the binary mixtures of BUA and THY molecules, which indicates a strong interaction between [Ch^+^][Cl^−^] salt and Butyric acid (see Fig. 6a).It should be noted that the average value of the distribution of hydrogen bonds between anions and BUA molecules was significantly greater compared to the very-long-chain fatty acids (DEC, MRA and etc.) (see Fig. 6b). The hydrogen bonds between fatty acid molecules were compared in the presence of thymol molecules and choline chloride salt at 353 K. The results show that there are strong H-bonds between HBA and HBD for the binary mixtures of [Ch^+^][Cl^−^] and FAs (on average 570.901 ± 0.6025 H-bond for the salt and DEC molecules). It was deduced from the persistence of hydrogen bond interactions between FAs molecules in the binary mixtures of THY and FAs molecules. However, the interaction between the –COOH group of FAs and the oxygen atom bonded to the aromatic ring of THYmolecule is the reducing agent of hydrogen bonding between FAs molecules in the binary mixtures of THY and FAs. The longer alkyl chain leads to reducing partial charges on the O atoms of FAs, allowing the ability of the H-bonds donor of FAs to minimize in the binary mixtures (see Fig. 6c). The strong hydrogen bond network existing between FAs and HBA is disrupted by the adjacent water molecules. The cause of the instability of eutectic solvents based on choline chloride and acids with alkyl chains of 4 carbons or longer is largely related to the local interactions such as hydrogen bonds between choline chloride and water molecules. It was found that, on average, each choline chloride salt receives ∼ 6.633 hydrogen bonds from water in the mixture of Ace and [Ch^+^][Cl^−^].

In addition, the average number of H-bonds between choline cation and chloride anion descend in the adjacent water. For the binary mixtures of Ace and [Ch^+^][Cl^−^], the average number of H-bonds between acid and water molecules was larger than 4 carbons in the binary mixtures of FAs and [Ch^+^][Cl^−^].

Hydrogen bonding interaction between Acetic acid and water was much more favorable (avg. 69.606 numbers) than Decanoic acid–water (avg. 23.739 numbers) and Myristic acid–water (avg. 6.633 numbers).

**Figure 7:**
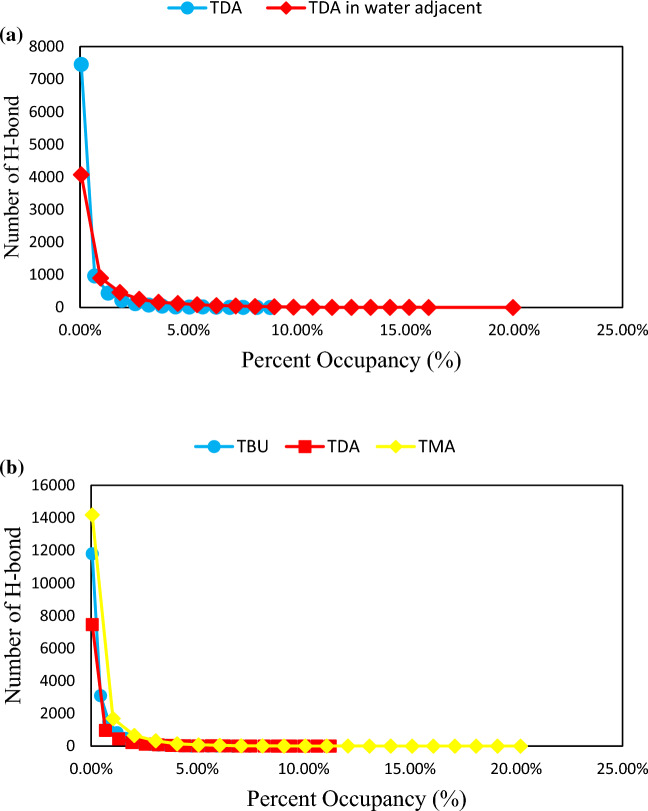
(**a**) Hydrogen bond percent occupancies for DEC and THY molecules for the binary mixtures in the adjacent water and the pure state. (**b**) Hydrogen bond percent occupancies for FAs and THY molecules for the binary mixtures in water adjacent at 353 K.

The long chain length of the fatty acid and the aromatic ring of thymol refers to the hydrophobic properties of the eutectic mixtures consisting of these two species, causing stability of DESs based on THY and FAs in the aqueous environment. However, the number of possible hydrogen bonds per molecule of acid was slightly reduced in the adjacent water. The relative percent occupancies of hydrogen bonds were taken to check the total time a unique hydrogen bond between species in during simulation^27^. The percent occupancies analysis showed that the stability hydrogen bond between HBA and HBD is one of the most significant interactions for the stability DES in water adjacent. The ability of H-bond donor atoms of FAs decreases with increasing the length of the alkyl chain. The negligible stability of hydrogen bonding between THY–water molecules (∼20%) in the mixture of TMA was determined at 353 K. There are hydrogen-bonding interactions between the THY molecules and FAs in water adjacent for longer times with occupancy of 7.46% and 11.25% in the mixtures of TBU, and TDA, respectively. This trend is confirmed by the viscosity of the binary mixtures at 353 K (see Fig. 7a,b).

### Non-bonded interaction energy and relative stability factor

To better understand the intermolecular interactions in the binary mixtures the non-bonded energy between species was computed. The nonbonding interaction energy includes the summation of electrostatic energy and van der Waals energy. E_vdW_ and E_Coul_ amounted to − 3.720 kcal/mol and − 93.48 kcal/mol for ${H}_{FAs}- {Cl}^{-}$ interaction in the binary mixture of Ace and [Ch^+^][Cl^−^] at 353 K. Clearly, the electrostatic interactions are more dominant than the vdW interactions during the simulation process. The results show a reduction in the energy of the non-bonding interactions between the anions and the HA atom of FAs with increasing the carbon chain length of the fatty acids. This reduction is related to electrostatic interactions. The results of changes in the binary mixtures of thymol and fatty acids are similar to the eutectic solvents based on fatty acids and choline chloride at a ratio of 1:1.

The electrostatic and van der Waals (vdW) interactions between the different species in the adjacent water were presented in Table 2. In this part of the article, we have compared the interaction energies between HBA and HBD in the adjacent water and the pure state. The interaction energies between the water molecules and fatty acids such as Acetic acid, Butyric acid, Decanoic acid, and Myristic acid were compared in the binary mixtures at 353 K. The interaction energy between fatty acids and the water molecules in the binary of Choline chloride and fatty acid has the following trend: Acetic acid–water (− 80.99 kcal/mol) > Butyric acid–water (− 49.55 kcal/mol) > Decanoic acid–water (− 9.422 kcal/mol) > Myristic acid–water (− 7.70 kcal/mol). Among the studied fatty acids, Acetic acid, which has the shortest alkyl chains, is the most effective of HBD in the interaction of the studied eutectic mixtures with water. The polar carboxyl (–COOH) group of fatty acids plays a key role in the formation of DES based on fatty acids that may easily interact with water molecules in aqueous environments. In general, HBA-HBD interaction in the thymol and fatty acid-based deep eutectic solvent based is more favorable than HBA-water interaction in comparison with the studied eutectic solvents. Broad affinities of HBA to the water-rich phase can simply be attributed to the hydrophilic nature of the eutectic solvents based on choline chloride salt. It should be noted that the two components of eutectic solvents based on thymol and fatty acids are nonionic and the electrostatic have a much smaller contribution compared to the mixtures containing choline chloride. The non-bonded interaction energy between choline chloride and FAs (− 97.2014 kcal/mol) for the binary mixtures of Ace and [Ch^+^][Cl^−^] decreased to (− 11.557 kcal/mol) in the adjacent water, whereas, ${E}_{total}$ between FAs and THY molecules has not changed much in the binary mixture. The effect of the HBA species on the stability of the eutectic mixtures based on fatty acids in the aqueous environment can be explained in terms of HBA – HBD interactions in the adjacent water. As expected, for the binary mixtures consisting of FAs and [Ch^+^][Cl^−^] salt, the lowest interaction energy value of − 7.6956 kcal/mol was observed for interaction between Myristic acid and water in binary mixtures with a ratio of 1:1. Similarly, the MRA and thymol molecules at the DES/water interface interact less with the water molecules in the thymol-fatty acids based deep eutectic solvent. In this work, we introduced the relative stability factor based on the non-bonded interaction energy. The relative Stability Factor is a relative measure of how much deep eutectic solvents are stable in aqueous environment. The stability factor primarily was considered as a function of the HBD–water interaction because the HBA–water interaction between donor and water is almost constant in the binary mixtures. This relative measure might be shown the relative stability of the eutectic solvents in the adjacent water, but only partially. The relative stability factor (S) is defined as

**Table 2:** MD simulated non-bonded interaction energies (kcal/mol) between the different pairs of HBA–HBD–water for the different systems calculated at 353 k.

System no	Component pairs	E_elec_	E_vdW_	E_total_	Relative stability factor (S)
CAC	HBA-HBD	− 0.193	− 10.5039	− 10.697	0.1063
	HBA-TIP3	0.3709	− 85.704	− 85.333	
	HBD-TIP3	− 0.5363	− 14.7486	− 15.285	
CBU	HBA-HBD	− 0.2005	− 10.529	− 10.729	0.1068
	HBA-TIP3	0.3726	− 85.886	− 85.5131	
	HBD-TIP3	− 0.5753	− 14.361	− 14.936	
CCA	HBA-HBD	− 0.2444	− 11.452	− 11.696	0.1157
	HBA-TIP3	0.3723	− 85.612	− 85.2402	
	HBD-TIP3	− 0.3351	− 15.525	− 15.86	
CDC	HBA-HBD	− 0.5962	− 12.387	− 12.984	0.1266
	HBA-TIP3	0.5438	− 83.977	− 83.433	
	HBD-TIP3	− 1.236	− 17.871	− 19.115	
CLA	HBA-HBD	− 0.4837	− 16.206	− 16.689	0.1402
	HBA-TIP3	0.532	− 86.477	− 85.945	
	HBD-TIP3	− 0.7201	− 32.384	− 33.104	
CMA	HBA-HBD	− 0.7004	− 11.588	− 12.289	0.3146
	HBA-TIP3	64.492	− 84.023	− 19.531	
	HBD-TIP3	− 1.799	− 17.732	− 19.531	
TAC	HBA-HBD	− 1.023	− 10.589	− 11.612	0.8734
	HBA-TIP3	− 0.923	− 2.771	− 3.694	
	HBD-TIP3	− 1.0111	− 8.591	− 9.6021	
TBA	HBA-HBD	− 2.9986	− 11.178	− 14.1762	1.371
	HBA-TIP3	− 0.807	− 2.504	− 3.311	
	HBD-TIP3	− 0.766	− 6.2641	− 7.0301	
TCA	HBA-HBD	− 5.058	− 21.571	− 26.629	3.7013
	HBA-TIP3	− 0.6349	− 2.088	− 2.723	
	HBD-TIP3	− 0.3605	− 4.111	− 4.472	
TDA	HBA-HBD	− 5.7264	− 21.592	− 27.319	4.0612
	HBA-TIP3	− 0.6434	− 2.0724	− 2.7159	
	HBD-TIP3	− 0.412	− 3.599	− 4.011	
TMA	HBA-HBD	− 6.1074	− 29.491	− 35.598	4.597
	HBA-TIP3	− 0.551	− 1.7479	− 2.298	
	HBD-TIP3	− 0.3624	− 5.082	− 5.444	

3 $$S=\frac{IE(HBA-HBD)}{IE \left(HBA-water\right)+IE(HBD-water)}$$

According to previous studies, the stability factor of the eutectic solvent ranging from 0 to 3.30 can be considered as solvent miscible in water^28^. In the case of the binary mixtures of FAs and choline chloride, the ranges for the stability factor are 0.1063 < S < 0.3146 at 353 K. (1) Eutectic solvents based on choline chloride and acids with short chain lengths are known as unstable solvents in water. (2) The partial stability of choline chloride-based eutectic solvents was increased with increasing carbon chain length. The assessment of the stability factor for the binary mixtures showed that thymol-based DESs, Decanoic acid (S = 4.0612), and Myristic acid (S = 4.597) were found to be the “most stable”. Interestingly, the stability of eutectic solvents based on choline chloride and FAs with alkyl chain lengths longer than 8 carbons is greater than that of the eutectic mixtures of the acetic acid and choline chloride (see Table 2).

### Spatial distribution function (SDF)

**Figure 8:**
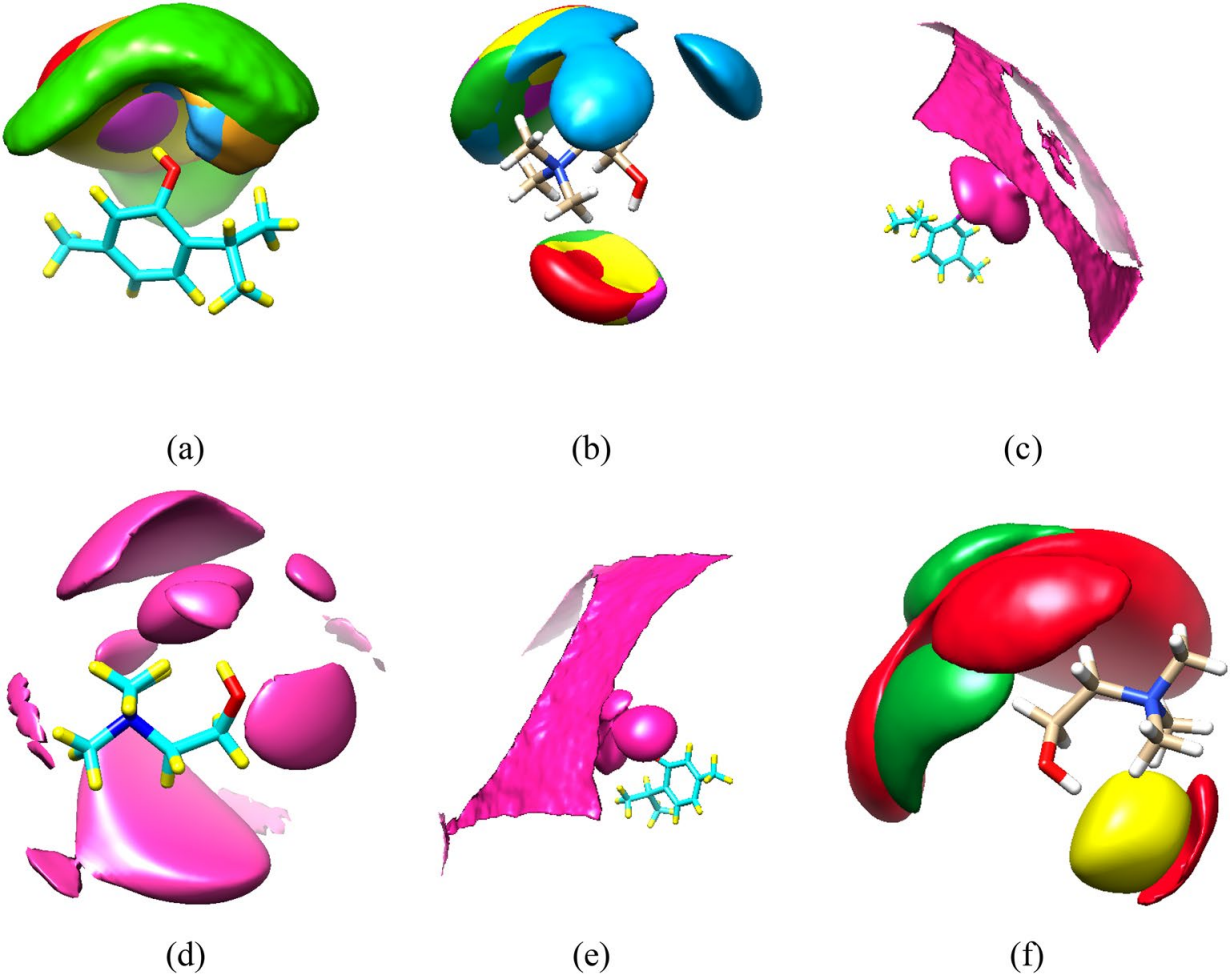
Spatial distribution functions (SDFs) of the eutectic mixture components with 50% FAs at 353 K. red isosurfaces correspond with choline cations, yellow isosurfaces are chloride anions, and green isosurfaces are FAs molecules, and Purple isosurfaces are water molecule.

The average density distribution of the water molecules around a reference molecule is illustrated by the SDF. The SDFs were calculated using the TRAVIS package^29^. SDFs of the HBA species around HBD molecules were discussed at 358 K. The SDF results suggest that the distribution of thymol molecules around the H atoms of the fatty acids alkyl chain is lower than the carboxyl (–COOH) group of FAs. In Fig. 8 one can also notice that the carboxyl (–COOH) group of fatty acids is surrounded by chloride anions in the pure DESs. Clearly, chloride anions are placed in the inner layer compared to cations. The molecule of fatty acids would prefer to interact with chloride anions rather than interact with the choline cations in the binary mixtures. Therefore, chloride anions play a key role in the formation of H-bonds between HBA and HBD in the eutectic solvents consisting of choline chloride and fatty acids. The three-dimensional density distribution of thymol molecules around FAs molecules is seen vividly, which decreases in the adjacent very long-chain fatty acids. To determine the significance of HBA species for the formation of a stable eutectic solvent in adjacent water, the spatial distribution function of water molecules around HBA species was investigated at 353 K. The purple isosurfaces is related to the densities of water molecules around THY molecules and choline cations in the binary mixtures. To construct the 3D isosurface, the particular density value (isovalues) was assumed to be equivalent to the completion of the first solvation shell. The calculated SDFs show that the water molecules are distributed around the active sites of HBA (-OH group of thymol and choline cation). However, the distribution of water molecules around the choline chloride salt is significant in the binary mixtures. As can be clearly seen in Fig. 8, Cl^−^ anions relative to a water molecule located in the first solvation shell and the choline cation in the second solvation (see Fig. 8a–f, Table S1).

### Thermo-physical properties analysis

The shear viscosity of the binary mixtures in molecular dynamics simulation was evaluated using the Green–Kubo method^30^. The following equation can be used for calculating the shear viscosity:

4 $$ {\upeta } = \frac{{\text{V}}}{{k_{B} T}}\mathop \smallint \limits_{0}^{\infty } dt < P_{xy} \left( 0 \right)P_{xy} \left( t \right) > , $$

where η is the shear viscosity, V and T represent the volume and the temperature of system, respectively. ${{\text{k}}}_{{\text{B}}}$ and ${P}_{xy}$ are Boltzmann constant and off-diagonal element of the stress tensor^31^. The calculated viscosity from simulation (η) is presented in Table 3. The viscosity values are about 1.8167, and 2.8148 (mPa s) for the mixtures of TBU, and TDA, respectively. The results of the simulation indicate that the long chain length of fatty acids makes an increase in the shear viscosity of the binary mixtures of THY and FAs at 353 K. The amount of the shear viscosity of the binary mixtures reaches from 1.5507 to 4.3142 mPa s with increasing the alkyl chain from C2 to C14. The results of particle density confirm the higher viscosity of the binary mixtures containing very long-chain fatty acids.

**Table 3:** The calculated self-diffusion coefficient ${\varvec{D}}\boldsymbol{ }({{\mathbf{A}}^{^\circ }}^{2}{\mathbf{n}\mathbf{s}}^{-1})$ of the species from the slope of the MSD plots for the binary mixtures at 353 K.

Systems	THY	Binary mixtures		Ch^+^	Cl^−^	FAs
		FAs	Systems			
TAC	3.73	27.56	CAC	0.098	0.01	1.48
TBU	7.42	26.7	CBU	0.13	0.076	0.6813
TDA	22.15	10.15	CDA	0.16	0.15	0.411
TMA	45.78	1.86	CMA	0.21	0.2064	0.013

### Molecule orientation

#### Vector reorientation dynamics

The reorientation of any the dipole vector was calculated via vector reorientation dynamics $VRD\left(\tau \right)$ analyses. It should be noted that the NMR experiment is also used to calculate the reorientation correlation times^25^.The autocorrelation of the vector can be defined as the normalized sum over the dot product between the vector at some time t and the same vector at some later time t + τ in the simulation box. $VRD\left(\tau \right)$ is given by,

5 $$ VRD\left( \tau \right) = N.\left\langle {\mathop \sum \limits_{t = 0}^{T - \tau } \vec{a}_{i} \left( t \right).\vec{a}_{i} \left( {t + \tau } \right)_{i} } \right\rangle $$

**Figure 9:**
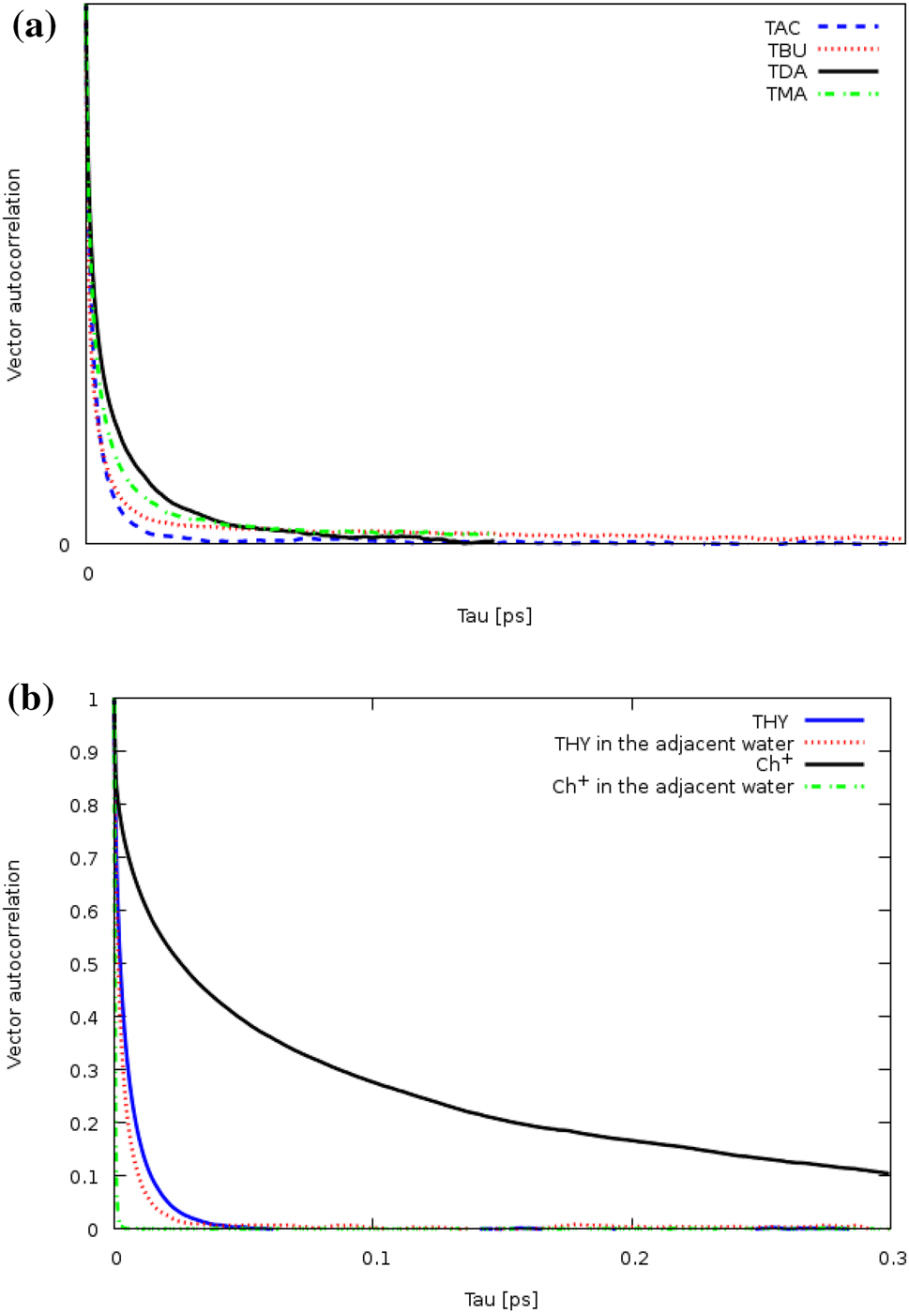
(**a**) Vector reorientation dynamics for O–H1 bond of THY for the binary mixtures of THY and FAs at 353 K. (**b**) Vector reorientation dynamics for the O1–H1 bond of Ch^+^ cation and THY molecule in the binary mixtures in adjacent water and pure state.

VRD(τ) is one criterion to specify the vector reorientation dynamics^32^. Curves of vector reorientation dynamics function typically start at value 1 and then fall to 0 with increasing the simulation time. The reorientation of the different vectors for species of thymol and [Ch^+^][Cl^−^] based DESs with fatty acids as HBD was investigated at 353 K. Reorientation curves of the C1–C2, C8–C9, and O–H bonds of THY molecules were depicted with dashed black, blue, and red lines, respectively. For thymol molecules, the orientation of the aromatic ring vectors is slower than that of the side chain (see Fig. S7). In the case of reorientation of vector O–H in the FAs، VRD(τ) function of the TMA mixture reaches this minimum value faster than other fatty acids. Faster orientation was possibly due to the weakening of intramolecular O–H⋯O hydrogen bonds (see Fig. 9a). The black and blue curves depict the reorientation of Vectors R1 and R2 in the pure state. Vectors R1 and R2 are shown in Fig. 9b. The hydroxyl group of choline cation is responsible for the intermolecular interaction in the binary mixtures, thus vector reorientation functions of O–H is slower than the hydroxyl group of thymol molecules. Some vector reorientation functions of HBA and HBD molecules in the adjacent water and pure state are shown in Fig. 8b. According to Fig. 9b, the reorientation of O–H bonds THY molecules and choline cation in the adjacent water is faster than the pure state. It should be noted that there is no clear trend in the vector reorientation dynamics of the H–O vector of water (see Fig. S8 and S9).

### The velocity autocorrelation functions (VACFs)

**Figure 10:**
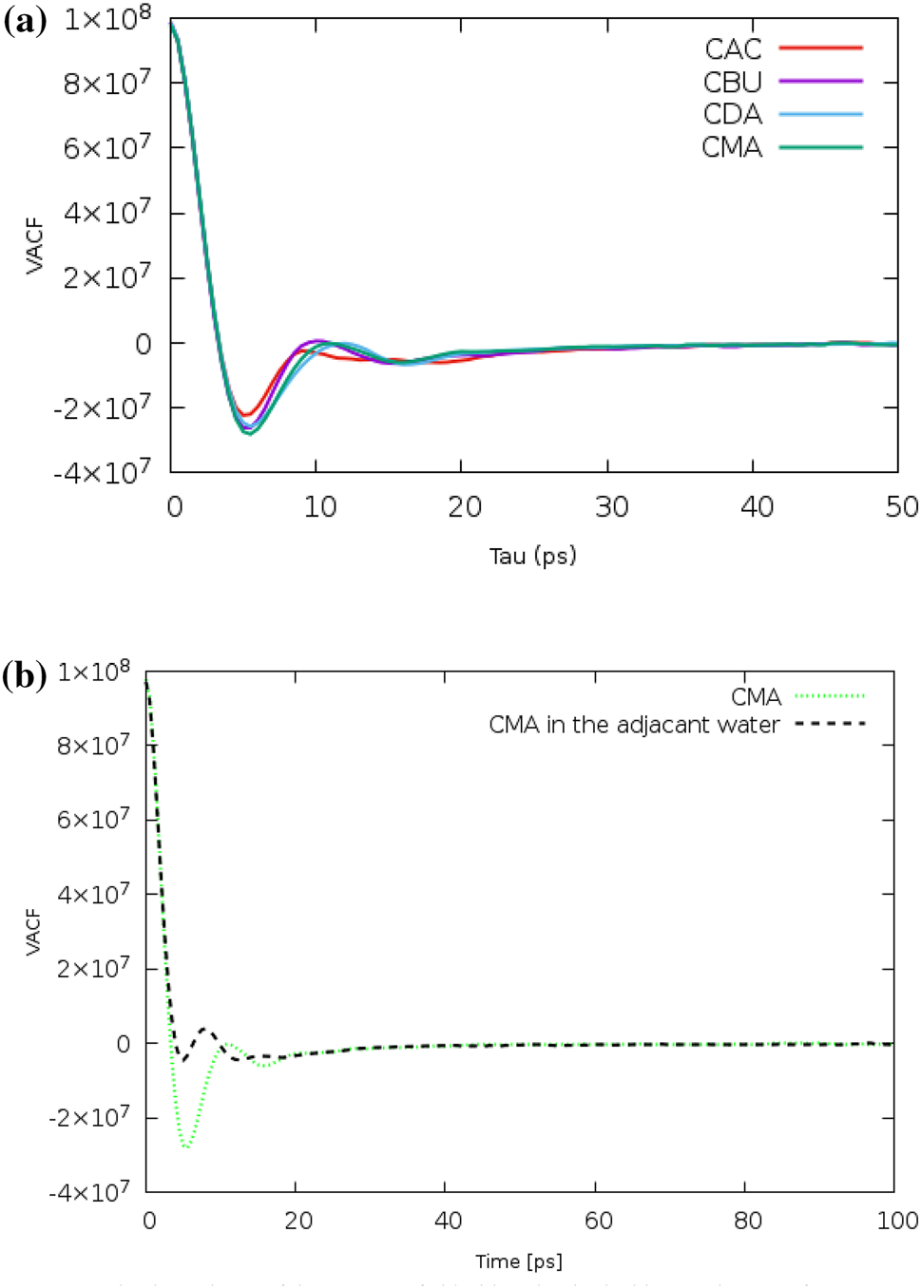
(**a**) The VACFs of chloride anions for the binary mixtures of FAs and [Ch^+^][Cl^−^] at 353 K. (**b**) The dependence of the VACFs of chloride anion in the binary mixtures of MRA and [Ch^+^][Cl^−^] at pure state and in adjacent water.

The dynamical behavior and microscopic motion of species in the binary mixtures can be analyzed by calculating the normalized velocity autocorrelation function (VACF)^33^. The mean collision time and the velocity randomization time of species can be estimated by these normalized VACF plots analysis^34^. The normalized VACF can be calculated by the relations:

6 $$ {\text{C}}\left( {\text{t}} \right) = \frac{{\left\langle {{\text{V}}_{{\text{i}}} \left( {\text{t}} \right).{\text{V}}_{{\text{i}}} \left( 0 \right)} \right\rangle }}{{\left\langle {{\text{V}}_{{\text{i}}} \left( 0 \right).{\text{V}}_{{\text{i}}} \left( 0 \right)} \right\rangle }} $$

where ${v}_{i}(t)$ are center-of-mass (COM) velocity vectors of species from recorded the 30 ns simulation trajectories. The brackets ⟨⟩ refer to the ensemble average all over the entire simulation time^35^. The VACFs of species deep eutectic solvents (DESs) based on different acids as hydrogen bond donors (HBDs) are compared in Fig. S10. The mean collision time and also velocity randomization time are calculated by the first zero and the second zero of the VACFs of species. The mean collision time for the Butyric acid in the binary mixture of [Ch^+^][Cl^−^] and BUA is estimated at around 20 ns while the value of this quantity for the TBU mixture is equal to 10 ns. Compared to the binary mixtures of THY and fatty acid, the mean collision time for species is enhanced in the Ch^+^/Cl^−^ mixtures. These results are in agreement with the intermolecular interactions. This means that the mean collision time (first zero) of species depends on the intermolecular interaction. It should be noted that the molecule collision time of FAs is shorter than the choline cation and chloride anion. The density of systems can be distinguished by examining the precise behavior of VACFs (see Fig. 10a). There is an interesting relationship suggesting a direct relationship between the depth well of the first minimum of VACF and the density of systems. There was a significant difference in VACF plots for condensed systems (Binary mixtures at pure state) and diluted systems (Binary mixtures in the adjacent water). The depth well of the first minimum of VACF is less such that the depth well is not detectable in dilution with water systems (see Fig. 10b). In the binary mixtures and the water/DES mixtures, the velocity randomization time of chloride anion is faster than other species. These observations are most likely related to the lower molar mass of anions in comparison with the ions.

### Rotational and lateral diffusion

**Figure 11:**
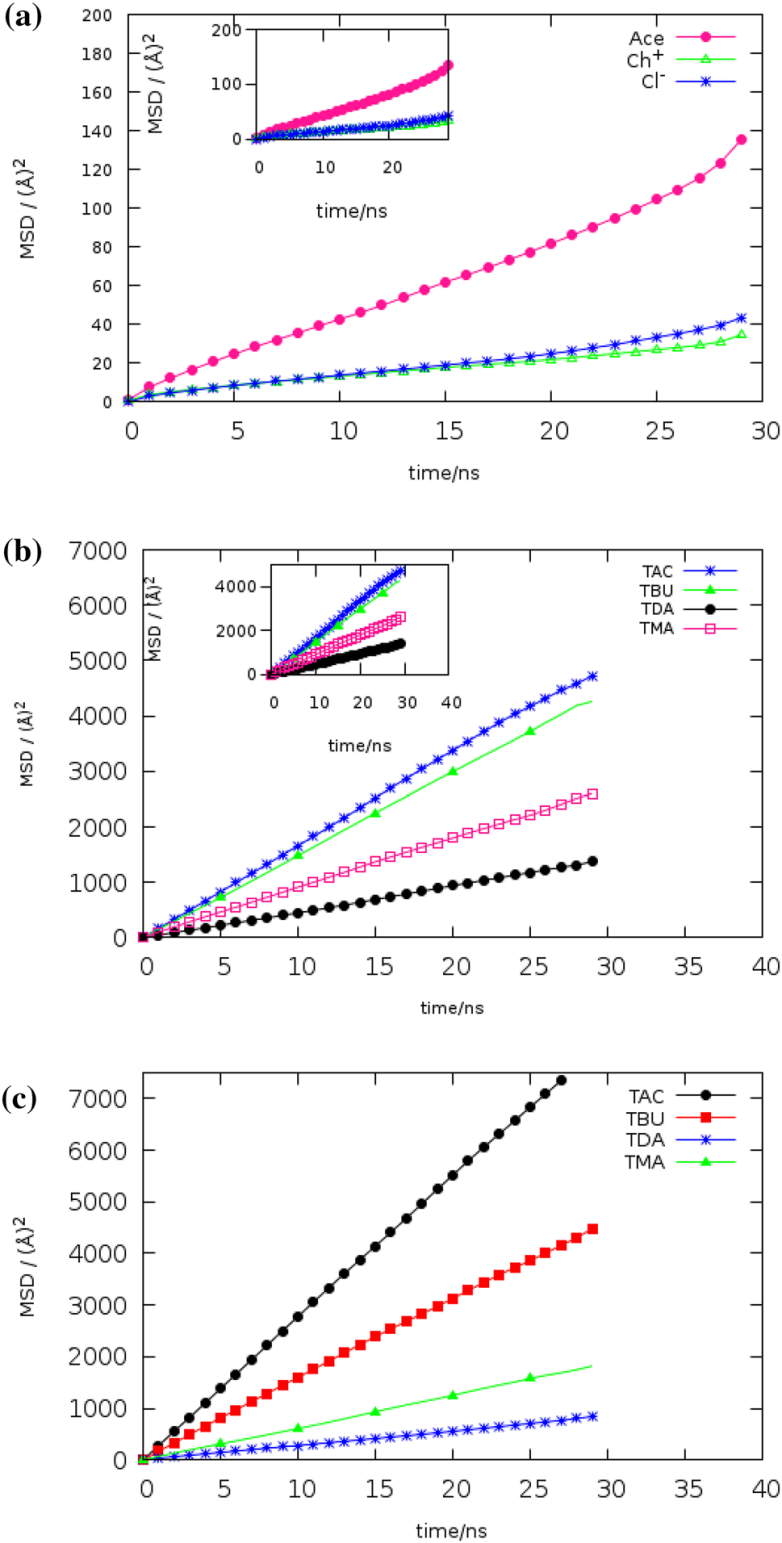
(**a**) The center of mass MSDs of Species of the binary mixture of Ace and [Ch^+^][Cl^−^] at 353 K. (**b**). The center of mass MSDs of THY molecules for the binary mixture at 353 K. (**c**) The center of mass MSDs of FAs molecules for the binary mixture at 353 K.

The means square displacement (MSDs) of HBA and HBD were investigated as a measure of the dynamic properties of the eutectic solvents at 353 K. MSDs of the center-of-mass for the different species in the [Ch^+^/Cl^−^] [FAs] and [THY][FAs] eutectic mixtures at a ratio of 1:1 were obtained from the 30 ns simulations. MSDs of the HBA species were plotted over time in the presence of different fatty acids (Fig. 11a–c). From all of the MSDs, the following outcomes can be expressed: (1) MSD of lighter FAs has a higher slope than the very long-chain fatty acids. (2) The slope of MSDs for cations, anions, and THY molecules is decreased in presence of the fatty acids with an alkyl chain length of fewer than 8 carbons. Reduction of the slope of MSDs of the ions and other species corresponds to intermolecular interactions. MSDs of HBA and HBD for the binary mixtures in the pure state and in adjacent water are presented in Fig. S11 (see Fig. S13). It is evident that the slope of Cl^−^ MSD in the binary mixture of BUA and Ch^+^/Cl^−^ is twice that of the Cl^−^ curve in the system containing Acetic acid. MSD of FAs molecules is dependent on the intermolecular interactions, which are different depending on the type of HBA in the binary mixtures. Reducing translational mobility of species is clearly demonstrated in pure state. Previous research has shown that the MSDs of species reach the hydrodynamic limit when β = 1. The results clearly show that the MSD of species in the binary mixtures reaches the diffusive regime (β ~ 1) after a long simulation time (30 ns). The self-diffusion coefficients of species were obtained from the slopes of the lines fitted to the MSD curves using the Einstein relation:

7 $$ Ds = \mathop {\lim }\limits_{t \to \infty } \frac{d}{dt}\frac{1}{6}\left\langle {[r_{i} \left( {\text{t}} \right) - r_{i} \left( 0 \right)]^{2} } \right\rangle $$

where $[{{r}_{i}\left({\text{t}}\right)-{r}_{i}\left(0\right)]}^{2}$ denotes mean square displacement (MSD) and bracket is the ensemble average^36^. The plot of β versus time for anions was shown in Fig. S12. According to previous discussions presented by Del Pópolo, the β parameter was calculated using the following equation:

8 $$ {\upbeta } = \frac{{d\log_{10} \left\langle {{\Delta r}(t)^{2} } \right\rangle }}{{d{\text{log}}_{10} t}} $$

The molecule is located in three regimes during the simulation, depending on the β parameter^37^. (1) β = 2: the inertial regime (at initial times), (2) β < 1: the sub-diffusive regime (at the intermediate time), (3) β = 1: the diffusive regime (at a long time). The values presented in Table 4 reveal that the self-diffusion coefficients of Cl^−^ anion in the mixture TMA are 2 times higher than that in the mixture TAC at 353 K. D_self_ of chloride anions for CAC, CBU, CDA and CMA systems at 353 K was demonstrated in Table 4. Self-diffusion value of simulated binary mixtures is in agreement with H-bond results. The self-diffusion coefficient of the center of mass of the THY molecules for the binary mixture of Ace and THY at a ratio of 1:1 is 3.73 Å^2^ ns^−1^ The low value of the Self-diffusion value of THY can be attributed to the greater interaction of HBA and HBD in the binary mixtures. D_self_ of the binary mixtures was plotted in Fig. S11 (see Fig. S12). Dynamical and structural properties in the binary mixtures were studied in the adjacent water and a pure state. The water molecules have been noted to play important role in the increasing self-diffusion coefficients of species. The values of the diffusion coefficient of other species observed for the other species also follow this trend except for systems containing Myristic acid which had the lowest change compared to the binary mixture at pure state.

**Table 4:** Particle density and the shear viscosity (η) of the binary mixtures from simulations.

System	Binary mixtures of FAs and THY	
	Shear viscosity (mPa s)	Particle density
TAC	1.5507	0.0618
TBU	1.8167	0.0637
TDA	2.8148	0.0916
TMA	4.3142	0.4773

## Conclusion

The growing demand for DESs as solvents in the solubility of drugs and removal of aqueous pollutants, a fundamental understanding of the stability of Deep Eutectic Solvents is indispensable. In this study, using MD simulation, dynamical and structural properties of the binary mixtures based on fatty acids at the ratio of 1:1 were investigated at 353 K. The results show that strong Ch^+^. O–H⋯–O. FAs and Cl^−^⋯H. FAs hydrogen bonds play an important role in the formation of eutectic solvents based on choline chloride and fatty acids. The electrostatic interaction and the hydrogen bond network between HBA and HBD of eutectic solvent is disrupted in the adjacent water. The thymol and FAs-based eutectic solvents are much more stable than that in the binary mixture based on FAs and [Ch^+^][Cl^−^].

### Supplementary Information

Supplementary Information.

## Data Availability

All data generated or analysed during this study are included in this published article [and its supplementary information files].

## References

[1] Metzger, R. Exposure to organic solvents causes neurologic diseases.

[2] Abbott AP, Boothby D, Capper G, Davies DL, Rasheed RK (2004). Deep eutectic solvents formed between choline chloride and carboxylic acids: versatile alternatives to ionic liquids. J. Am. Chem. Soc..

[3] Sarmad S, Mikkola JP, Ji X (2017). Carbon dioxide capture with ionic liquids and deep eutectic solvents: A new generation of sorbents. Chemsuschem.

[4] Hayyan M, Looi CY, Hayyan A, Wong WF, Hashim MA (2015). In vitro and in vivo toxicity profiling of ammonium-based deep eutectic solvents. PLoS ONE.

[5] Gu T, Zhang M, Tan T, Chen J, Li Z, Zhang Q (2014). Deep eutectic solvents as novel extraction media for phenolic compounds from model oil. Chem. Commun..

[6] Shishov A, Pochivalov A, Nugbienyo L, Andruch V, Bulatov A (2020). Deep eutectic solvents are not only effective extractants. TrAC Trends Anal. Chem..

[7] Aissaoui T, AlNashef IM, Qureshi UA, Benguerba Y (2017). Potential applications of deep eutectic solvents in natural gas sweetening for CO_2_ capture. Rev. Chem. Eng..

[8] Kumar N, Naik PK, Banerjee T (2022). Molecular dynamic insights into the distinct solvation structures of aromatic and aliphatic compounds in monoethanolamine-based deep eutectic solvents. J. Phys. Chem. B.

[9] van Osch DJ, Zubeir LF, van den Bruinhorst A, Rocha MA, Kroon MC (2015). Hydrophobic deep eutectic solvents as water-immiscible extractants. Green Chem..

[10] McGaughy K, Reza MT (2020). Liquid-liquid extraction of furfural from water by hydrophobic deep eutectic solvents: improvement of density function theory modeling with experimental validations. ACS Omega.

[11] Zhu W, Wang C, Li H, Wu P, Xun S, Jiang W (2015). One-pot extraction combined with metal-free photochemical aerobic oxidative desulfurization in deep eutectic solvent. Green Chem..

[12] Homan T, Shahbaz K, Farid MM (2017). Improving the production of propyl and butyl ester-based biodiesel by purification using deep eutectic solvents. Sep. Purif. Technol..

[13] Florindo C, Branco L, Marrucho I (2017). Development of hydrophobic deep eutectic solvents for extraction of pesticides from aqueous environments. Fluid Phase Equilib..

[14] Klaiber A, Kollek T, Cardinal S, Hug N, Drechsler M, Polarz S (2018). Electron transfer in self-assembled micelles built by conductive polyoxometalate-surfactants showing battery-like behavior. Adv. Mater. Interfaces..

[15] Zurob E, Cabezas R, Villarroel E, Rosas N, Merlet G, Quijada-Maldonado E (2020). Design of natural deep eutectic solvents for the ultrasound-assisted extraction of hydroxytyrosol from olive leaves supported by COSMO-RS. Sep. Purif. Technol..

[16] Martínez L, Andrade R, Birgin EG, Martínez JM (2009). PACKMOL: A package for building initial configurations for molecular dynamics simulations. J. Comput. Chem..

[17] Barani Pour S, Sardroodi JJ, Ebrahimzadeh AR (2022). Structure and dynamics of hydrophobic deep eutectic solvents composed from terpene-fatty acids investigated by molecular dynamics simulation. J. Mol. Graph. Modell..

[18] Nam K, Gao J, York DM (2005). An efficient linear-scaling Ewald method for long-range electrostatic interactions in combined QM/MM calculations. J. Chem. Theory Comput..

[19] de Leeuw SW, Perram JW, Smith ER (1980). Simulation of electrostatic systems in periodic boundary conditions. I. Lattice sums and dielectric constants. Proc. R. Soc. Lond. A Math. Phys. Sci..

[20] Omelyan IP (1998). Algorithm for numerical integration of the rigid-body equations of motion. Phys. Rev. E.

[21] Mendez-Morales T, Carrete J, Bouzon-Capelo S, Perez-Rodriguez M, Cabeza O, Gallego LJ (2013). MD simulations of the formation of stable clusters in mixtures of alkaline salts and imidazolium-based ionic liquids. J. Phys. Chem. B.

[22] Zhu Y, Su L, Chen M, Su Y, Ji X, Gui X (2015). Controlled growth of epitaxial wurtzite BeMgZnO alloy films and two microscopic origins of Be–Mg mutual stabilizing mechanism. J. Alloys Compd..

[23] Morita M, Oya Y, Kato N, Mori K, Koyanagi J (2022). Effect of electrostatic interactions on the interfacial energy between thermoplastic polymers and graphene oxide: a molecular dynamics study. Polymers.

[24] Xu H, Kong Y, Peng J, Wang W, Li B (2021). Mechanism of deep eutectic solvent delignification: insights from molecular dynamics simulations. ACS Sustain. Chem. Eng..

[25] Barani Pour S, Sardroodi JJ, Ebrahimzadeh AR (2022). Structure and dynamics of thymol-fatty acids based deep eutectic solvent investigated by molecular dynamics simulation. Fluid Phase Equilib..

[26] Jahanbin Sardroodi J, Rastkar Ebrahimzadeh A, Avestan MS (2022). Structural and dynamic properties of eutectic mixtures based on menthol and fatty acids derived from coconut oil: A MD simulation study. Sci. Rep..

[27] Barani Pour S, Sardroodi JJ, Ebrahimzadeh AR (2021). The study of structure and interactions of glucose-based natural deep eutectic solvents by molecular dynamics simulation. J. Mol. Liq..

[28] Paul N, Naik PK, Ribeiro BD, Gooh Pattader PS, Marrucho IM, Banerjee T (2020). Molecular dynamics insights and water stability of hydrophobic deep eutectic solvents aided extraction of nitenpyram from an aqueous environment. J. Phys. Chem. B..

[29] Frömbgen T, Blasius J, Alizadeh V, Chaumont A, Brehm M, Kirchner B (2022). Cluster analysis in liquids: A novel tool in TRAVIS. J. Chem. Inform. Model..

[30] Barani Pour S, Jahanbin Sardroodi J, Rastkar Ebrahimzadeh A, Sadegh Avestan M (2022). Using molecular dynamics simulations to understand the effect of fatty acids chain length on structural and dynamic properties of deep eutectic solvents based on choline chloride and fatty acids. ChemistrySelect.

[31] Nevins D, Spera F (2007). Accurate computation of shear viscosity from equilibrium molecular dynamics simulations. Mol. Simul..

[32] Barani Pour S, Behrooz NJ, Sardroodi JJ, Ebrahimzadeh AR, Pazuki GR (2023). Differences in the effect of water on deep eutectic solvents based on choline chloride/lauric acid and choline chloride/myristic acid: molecular dynamics simulations at three different temperatures. ChemistrySelect.

[33] Kowsari MH, Tohidifar L (2016). Tracing dynamics, self-diffusion, and nanoscale structural heterogeneity of pure and binary mixtures of ionic liquid 1-hexyl-2, 3-dimethylimidazolium bis (fluorosulfonyl) imide with acetonitrile: Insights from molecular dynamics simulations. J. Phys. Chem. B.

[34] Wang Y-L, Shimpi MR, Sarman S, Antzutkin ON, Glavatskih S, Kloo L (2016). Atomistic insight into tetraalkylphosphonium bis (oxalato) borate ionic liquid/water mixtures. 2. Volumetric and dynamic properties. J. Phys. Chem. B.

[35] Srinivasan H, Sharma V, Mitra S, Biswas R, Mukhopadhyay R (2019). Dynamics in acetamide+ LiNO_3_ deep eutectic solvents. Phys. B Condens. Matter..

[36] Pranami G, Lamm MH (2015). Estimating error in diffusion coefficients derived from molecular dynamics simulations. J. Chem. Theory Comput..

[37] Pour SB, Sardroodi JJ, Ebrahimzadeh AR (2023). Effect of water addition on caprylic acid: Quaternary ammonium salts (QAS) deep eutectic solvents: Characterization of their structural and dynamical properties. J. Mol. Graph. Modell..

