# Supplementary material for: A comparative study of deep eutectic solvents based on fatty acids and the effect of water on their intermolecular interactions
Source: Sci Rep. 2024 Jan 19;14:1763. doi: 10.1038/s41598-023-50766-1 (PMC10799040; doi:10.1038/s41598-023-50766-1)
Supplement: Supplementary file 1 — Supplementary Information. [file 41598_2023_50766_MOESM1_ESM.docx]

Table S1. Coordination Numbers of molecules around each other in the binary mixtures at 353 K

|  | HBA----HBD | | HBA----HBD | |
| --- | --- | --- | --- | --- |
|  | Binary mixtures | | Binary mixtures in adjacency water | |
| system | r (Å) | Ncoor | r (Å) | Ncoor |
| CAC | 2.15 | 0.495 | 2.17 | 0.0095 |
| CBU | 2.15 | 0.4651 | 2.17 | 0.0101 |
| CDA | 2.15 | 0.3541 | 2.17 | 0.0278 |
| CMA | 2.15 | 0.1485 | 2.17 | 0.04 |
| TAC | 2.15 | 0.3902 | 2.17 | 0.0277 |
| TBU | 2.15 | 0.1639 | 2.17 | 0.0517 |
| TDA | 2.15 | 0.1486 | 2.17 | 0.0978 |
| TMA | 2.15 | 0.1409 | 2.17 | 0.2191 |

Table S2. The average number of hydrogen bonds between the species (N_avg_) in the binary mixtures.

| HBA – HBD | HBA – HBD | | | System |
| --- | --- | --- | --- | --- |
| Binary Mixtures in adjacency water | | | Binary Mixtures |  |
| - | | - | | CAC |
| - | | 684.438 +/- 0.5573 | | CBU |
| - | | 570.901 +/- 0.6025 | | CDA |
| - | | 491.525 +/- 1.068 | | CMA |
| 116.174 +/- 0.5596 |  | | |  |
| 231.473 +/- 0.6212 | 400.457 +/- 0.6986 | | | TAC |
| 291.051 +/- 0.5735 | 380.906 +/- 0.5956 | | | TBU |
| 358.452 +/- 0.9577 | 454.647 +/- 0.7165 | | | TDA |
| - | - | | | TMA |

| Acetic acid | 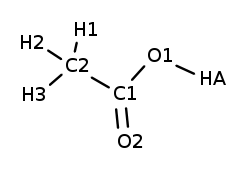 |
| --- | --- |
| Butyric acid | 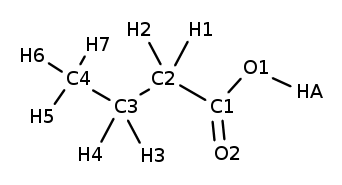 |
|  |  |
| Decanoic acid | 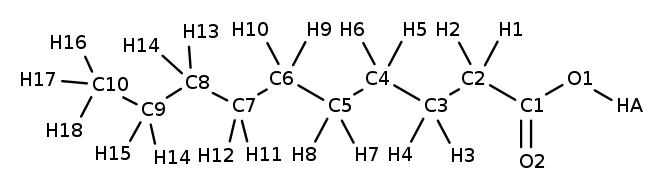 |
|  |  |
| Myristic acid | 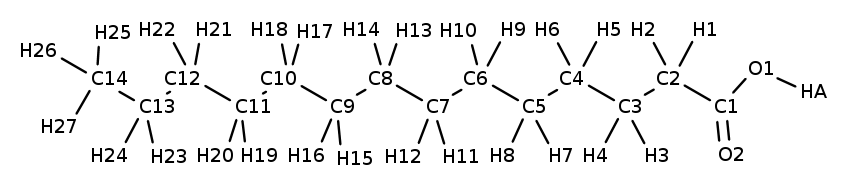 |
| Thymol | 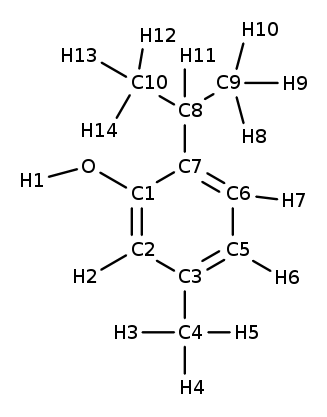 |
| Choline Chloride | 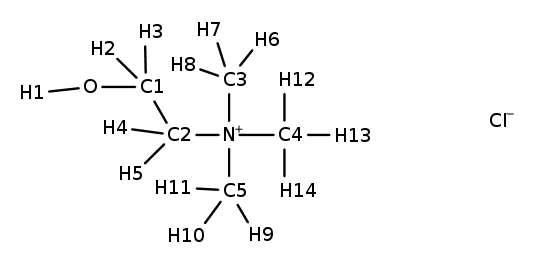 |
| Fig.S1. Schematic of Thymol (THY), fatty acids (FAs) and Choline Chloride (Ch^+^/Cl^-^) with the main atomic labels. | |


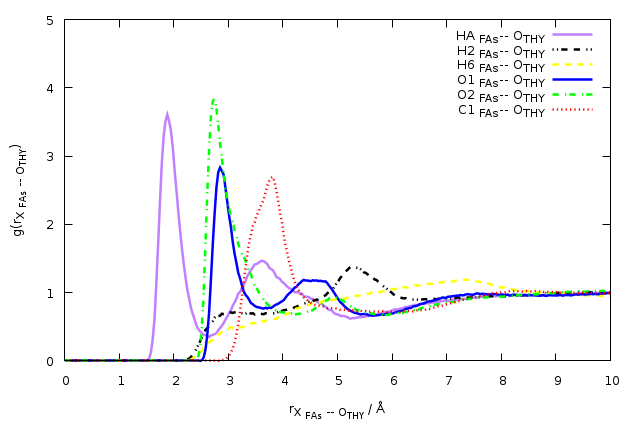


Fig. S2. Site-site RDFs between the different atoms of CAP and the O atom of Cl^-^ anion for the binary mixtures at a ratio of 1:1 at 353 K.


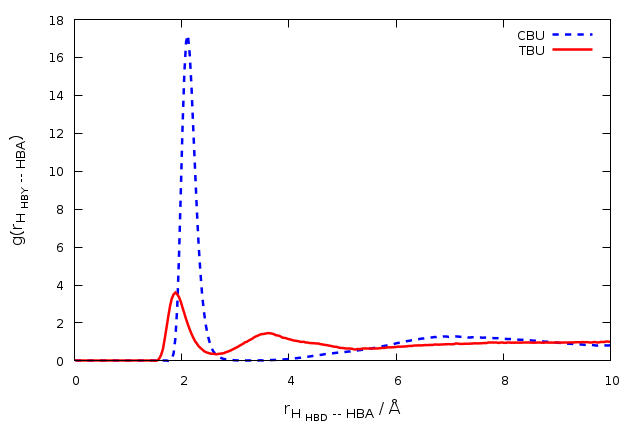


Fig.S3. RDFs between FAs molecules and HBA, $g(r_{HBD--HBA})$, for the binary mixture at 353 K.


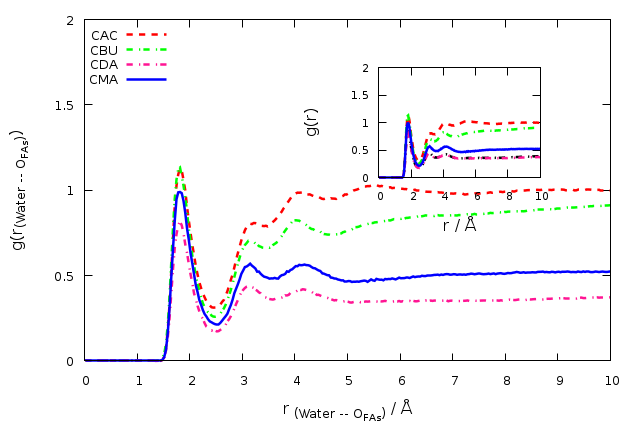


Fig. S4. RDFs between the HA atom of water molecules and the O atom of FAs molecules,${g(r)}_{Water - FAs}$, for the binary mixture of FAs and choline chloride at the adjacent water.


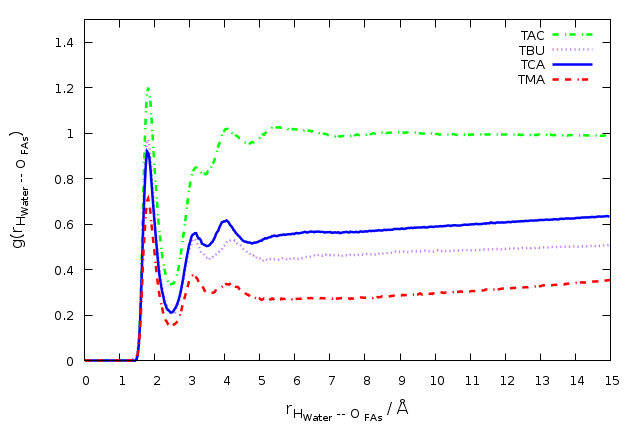


Fig. S5. RDFs between the HA atom of water molecules and the O atom of THY molecules,${g(r)}_{Water - THY}$, for the binary mixture at the adjacent water.

| 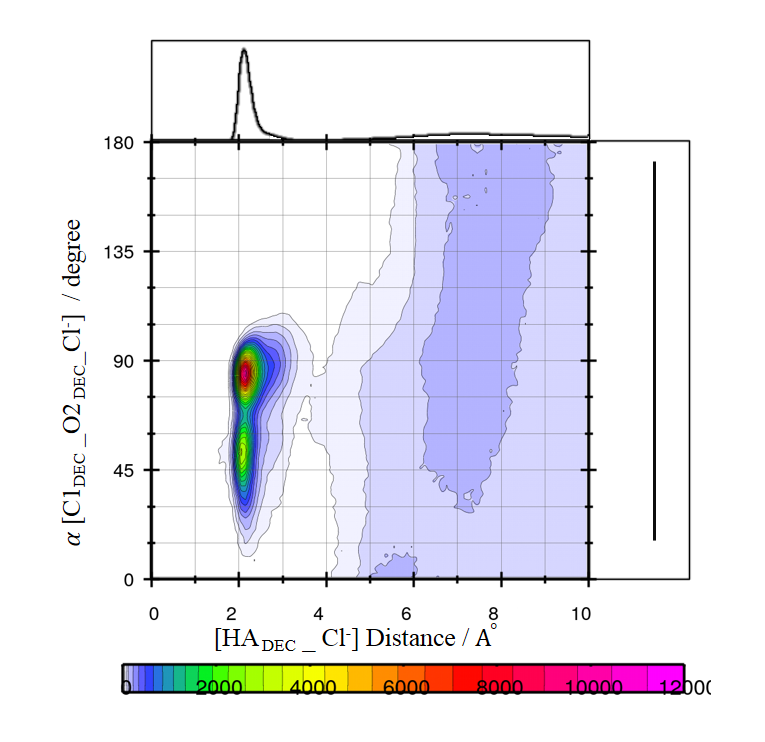 |
| --- |
| Fig. S6. Combined radial/angular distribution functions for the HA DEC _ Cl^-^ distance and C1DEC_O2 DEC_Cl^-^ angle. |
| 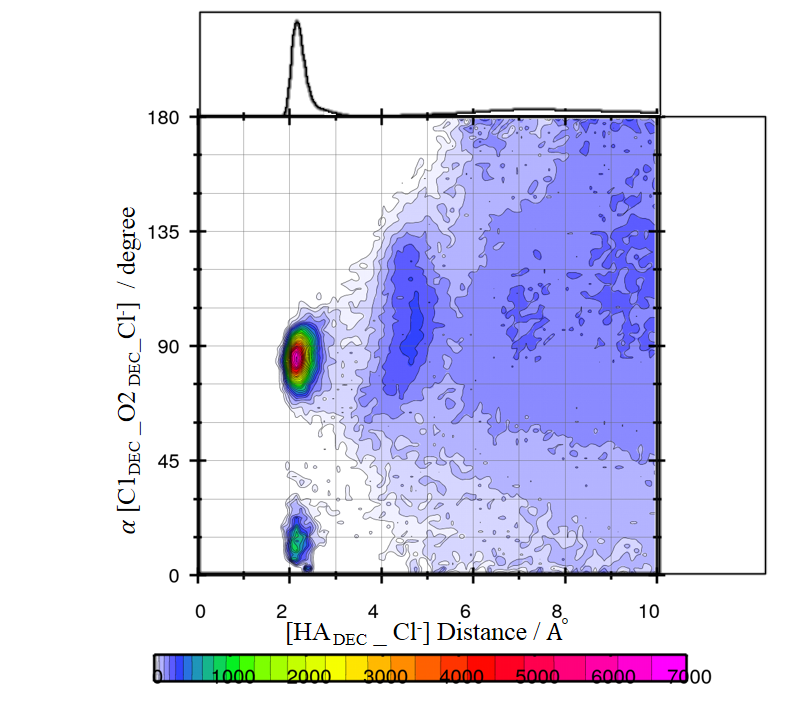 |
| Fig. S7. Combined radial/angular distribution functions for the HA DEC _ Cl^-^ distance and C1DEC_O2 DEC_Cl^-^ angle in the adjacent water. |


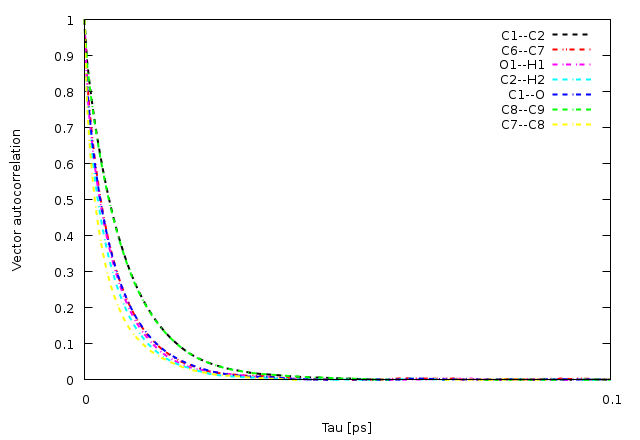


Fig. S8. Vector reorientation dynamics for different bonds of THY for the binary mixtures of FAs and THY at 353 K.


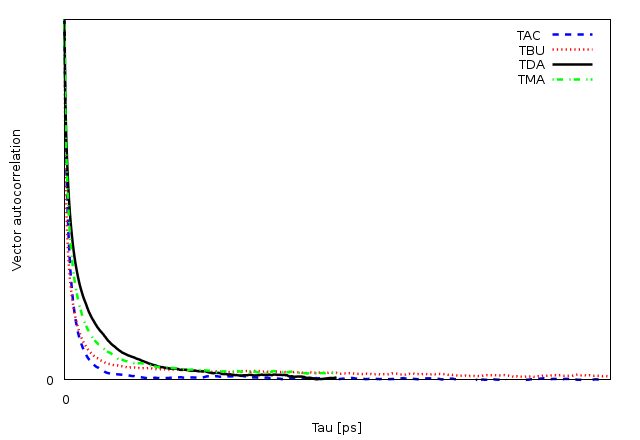


Fig. S9. Vector reorientation dynamics for O1—H1 bond of THY for the binary mixtures of FAs and THY in the adjacent water.


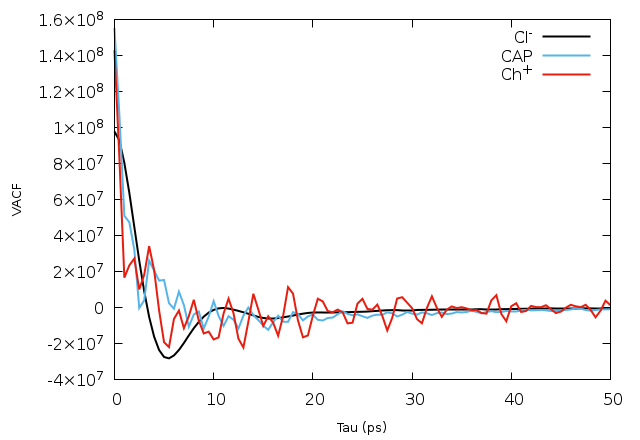


Fig. S10. The calculated VACF for the center of mass of [Ch^+^] and CAP molecule and [Cl^-^] anions in the binary mixture at 353 K.


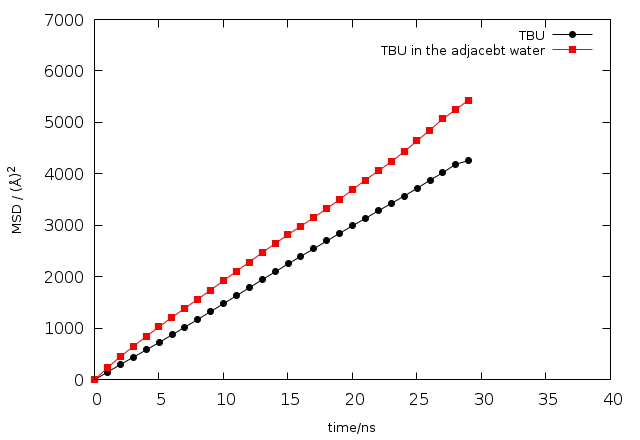


Fig.S11. The center of mass MSDs of Ch^+^ cation for the binary mixture at pure state and the adjacent water.

Fig.S12. The self-diffusion coefficient of Chloride anions and Choline cation of the binary mixtures at 353 K.

|  |
| --- |
| Fig.S13. The values of β during 30 ns of MD simulation for the chloride anions at 353 K. |


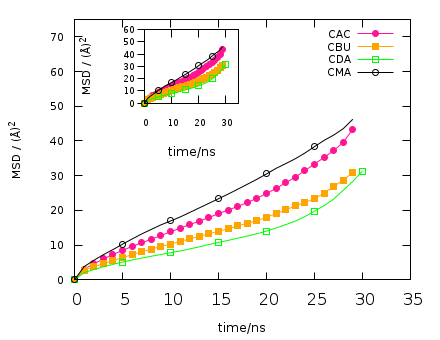


Fig.S14. The center of mass MSDs of Cl^-^ anion for the binary mixture at 353 K.


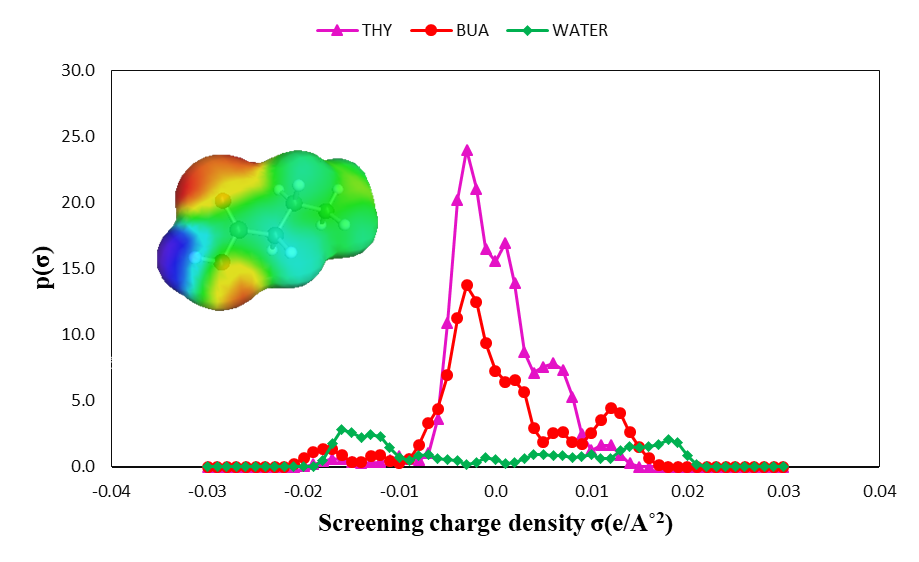

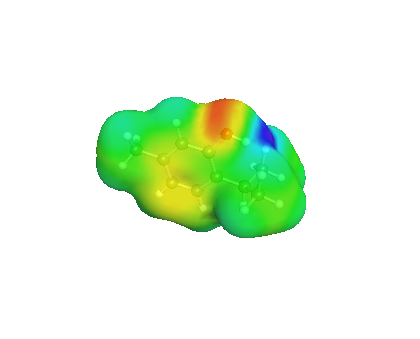

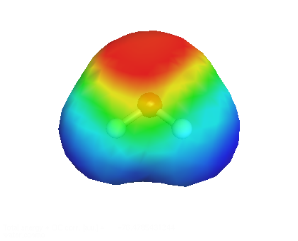


**H-bond acceptor region**

**Non-polar region**

**H-bond donor region**

References:

Fig. S15. Sigma - profiles of thymol (red), Butyric acid (pink), and water (green) and their sigma-surfaces representation.
